# Respiration supports intraphagosomal filamentation and escape of *Candida albicans* from macrophages

**DOI:** 10.1128/mbio.02745-23

**Published:** 2023-12-01

**Authors:** Nicola T. Case, Johannes Westman, Michael T. Hallett, Jonathan Plumb, Aiman Farheen, Michelle E. Maxson, Jessie MacAlpine, Sean D. Liston, Bernhard Hube, Nicole Robbins, Luke Whitesell, Sergio Grinstein, Leah E. Cowen

**Affiliations:** 1 Department of Molecular Genetics, University of Toronto, Toronto, Ontario, Canada; 2 Program in Cell Biology, Peter Gilgan Centre for Research and Learning, The Hospital for Sick Children, Toronto, Ontario, Canada; 3 Department of Biochemistry, Western University, London, Ontario, Canada; 4 Program in Cell Biology, The Hospital for Sick Children, Toronto, Ontario, Canada; 5 Department of Microbial Pathogenicity Mechanisms, Leibniz Institute for Natural Product Research and Infection Biology, Hans Knoell Institute, Jena, Germany; 6 Institute of Microbiology, Friedrich Schiller University, Jena, Germany; 7 Department of Biochemistry, University of Toronto, Toronto, Ontario, Canada; 8 Keenan Research Center of the Li Ka Shing Knowledge Institute, St. Michael’s Hospital, Toronto, Ontario, Canada; Yonsei University, Seoul, South Korea

**Keywords:** hyphae, morphogenesis, fungi, host-pathogen interactions, phagosome, glucose

## Abstract

For the human fungal pathogen *Candida albicans*, metabolic flexibility and the ability to transition between yeast and filamentous growth states are key virulence traits that enable disease in the host. These traits are particularly important during the interaction of *C. albicans* with macrophages, where the fungus must utilize multiple alternative carbon sources to survive after being phagocytosed, and filamentation is coupled to fungal escape and immune cell death. Here, we employed functional genomic screening of conditional-expression mutants covering >50% of the *C. albicans* genome to identify genes selectively required for filamentation inside macrophages. Through manual and machine learning-based image analyses, we uncovered a role for the mitochondrial ribosome, respiration, and the SNF1 AMP-activated kinase complex in governing filamentous growth within the phagosome, suggesting that *C. albicans* relies on respiration to evade the antifungal activities of macrophages. We demonstrate that downregulating the expression of these genes reduces ATP levels and impedes filamentation as well as growth under monoculture conditions in medium lacking glucose. In co-culture with physiological glucose concentration, downregulation of genes involved in mitochondrial function and respiration prevented *C. albicans* from expanding within the phagosome, escaping, and inducing immune cell death. Together, our work provides new insights into the impact of metabolism on the interaction between *C. albicans* and macrophages, highlighting respiration and the SNF1 AMP-activated kinase as key effectors of *C. albicans* metabolic flexibility and filamentation within phagocytes.

**IMPORTANCE:**

*Candida albicans* is a leading human fungal pathogen that often causes life-threatening infections in immunocompromised individuals. The ability of *C. albicans* to transition between yeast and filamentous forms is key to its virulence, and this occurs in response to many host-relevant cues, including engulfment by host macrophages. While previous efforts identified *C. albicans* genes required for filamentation in other conditions, the genes important for this morphological transition upon internalization by macrophages remained largely enigmatic. Here, we employed a functional genomic approach to identify genes that enable *C. albicans* filamentation within macrophages and uncovered a role for the mitochondrial ribosome, respiration, and the SNF1 AMP-activated kinase complex. Additionally, we showed that glucose uptake and glycolysis by macrophages support *C. albicans* filamentation. This work provides insights into the metabolic dueling that occurs during the interaction of *C. albicans* with macrophages and identifies vulnerabilities in *C. albicans* that could serve as promising therapeutic targets.

## INTRODUCTION

Fungi pose a major threat to human health, infecting over a billion people and claiming 1.5 million lives worldwide annually (1). *Candida albicans* is a member of the normal human mucosal microbiota, where, as an opportunistic pathogen, it can cause diseases ranging from superficial infections to life-threatening systemic candidiasis in immunocompromised individuals, with mortality rates upward of 40% (1, 2). *C. albicans* virulence is enabled in part by its ability to transition between yeast and filamentous growth states. The yeast form is associated with colonization and is crucial for dissemination via the bloodstream, whereas filamentation is important for tissue invasion and deep-seated infection (3). Although the relationship between morphogenesis and virulence is complex, the majority of *C. albicans* mutants locked in either state are avirulent in mouse models of systemic infection (4–8).

Phagocytes, such as macrophages, are among the first line of defense against fungal infections (9). Macrophages readily engulf *C. albicans* yeast cells, but the fungus can survive and filament within the maturing phagolysosome (10). Filamentation is coupled to cell wall remodeling, which induces proinflammatory cell death (pyroptosis) of the macrophage during the first 8 h post-phagocytosis, often referred to as Phase I death, and fungal escape (11–13). Approximately 9 h post-infection, Phase II death is initiated, during which *C. albicans* cells, which have escaped largely through physical rupture of the phagosome, further deplete glucose from the microenvironment surrounding the phagocyte, triggering macrophage cell death (11, 14, 15). The filament-specific peptide toxin candidalysin contributes to host cell killing in both phases (16).

Previous studies have used proteomic and transcriptomic approaches to study the response of *C. albicans* to phagocytosis by macrophages (14, 17, 18). These analyses revealed that, upon internalization, *C. albicans* rapidly shifts its metabolism from glycolysis toward respiration and the utilization of multiple alternative (non-fermentable) carbon sources such as carboxylic acids, amino acids, and *N*-acetylglucosamine (GlcNAc) (14, 17, 18). After *C. albicans* has escaped and Phase II death is initiated, the fungus reverts its metabolism, and cells quickly resume glycolytic growth (14, 17). Construction of *C. albicans* mutants lacking genes important for metabolism of amino acids, carboxylic acids, and GlcNAc demonstrated that disruption of any one of the aforementioned alternative metabolic pathways results in little impairment of intraphagosomal filamentation of *C. albicans* and modest to no attenuation of virulence in macrophages and in mouse models of infection (19–23). Rather, deletion of genes in all three pathways was needed to block filamentation in macrophage co-cultures and impair virulence in mice (23). Overall, the genetic circuitry that underlies intraphagosomal filamentation is complex, and much remains to be learned.

To gain further insights, we used a functional genomic approach to identify genes that, when transcriptionally repressed, impair intraphagosomal filamentation of *C. albicans*. To do so, we leveraged the gene replacement and conditional expression (GRACE) library, which covers >50% of the *C. albicans* nuclear genome and consists of mutants in which one allele of a target gene is deleted and the remaining allele is controlled by a tetracycline-repressible promoter (24, 25). In these strains, the addition of the tetracycline analog doxycycline (DOX) represses expression of the remaining wild-type allele (24, 25). Through image-based arrayed screening of the GRACE library under standard tissue culture (TC) conditions in the presence and absence of phagocytes, we identified 318 genes required for intraphagosomal filamentation but dispensable for filamentation under monoculture conditions. Among these genes, we found those encoding key components of the mitochondrial ribosome, respiratory machinery, and the SNF1 AMP-activated kinase (AMPK) complex in *C. albicans* to be essential in enabling fungal growth and filamentation in macrophages, phagosome escape, and phagocyte killing. Overall, the results reported here provide new biological insights into the regulation of intraphagosomal filamentation and identify vulnerabilities in *C. albicans* that could serve as promising targets for the future development of anti-virulence therapeutics.

## RESULTS

### Functional genomic screen identifies mutants unable to filament in phagocytes

**FIG 1:**
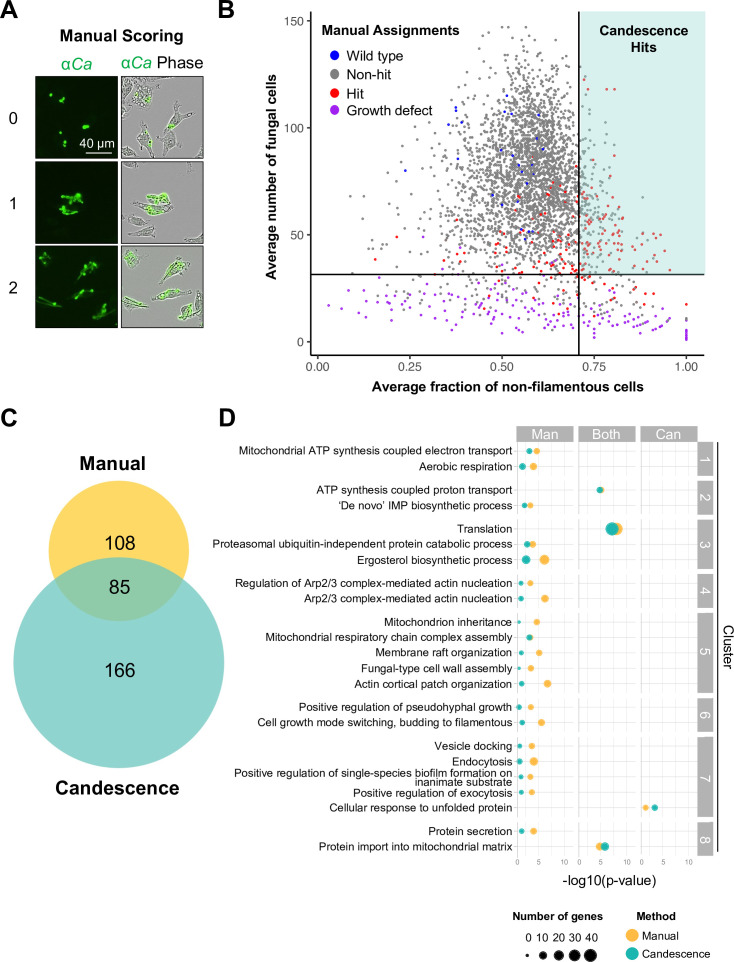
Functional genomic screen identifies *C. albicans* genes required for filamentation in phagocytes. (**A**) The degree of filamentation for each GRACE strain was manually scored after co-culture with mouse monocyte-macrophage lineage J774A.1 cells (MΦ). GRACE strains were grown overnight in the presence of a low concentration of doxycycline (low DOX, 0.05 µg/mL) prior to co-culture with MΦ cells for 4 h in the presence of a higher concentration of DOX (high DOX, 5 µg/mL) to maximally repress gene expression. Co-cultures were then fixed, permeabilized, and stained with FITC-conjugated anti-*C*. *albicans* antibody (α*Ca*, green signal). Representative images of a strain from each manual scoring category are shown. (**B**) Plot depicting genes required for filamentation in phagocytes as determined by MΦ-Candescence (turquoise-shaded quadrant). The *x*-axis indicates the average fraction of non-filamentous cells in MΦ images across both replicates for each GRACE strain. The *y*-axis indicates the geometric average of the number of internalized *C. albicans* cells across both replicates, as determined computationally by MΦ-Candescence. The vertical line marks the 90th quantile, with positive MΦ-Candescence predictions defined as those strains lying to the right of this line. The horizontal line marks the 95th quantile of all strains manually labeled as having a growth defect (purple dots). Blue dots correspond to wild-type control strains included on multiple GRACE library plates; red dots correspond to strains identified as hits by manual analysis; and gray dots correspond to all other strains that were not determined to be hits by manual annotation. The turquoise-shaded quadrant indicates strains impaired in their ability to filament in phagocytes, as determined by MΦ-Candescence. (**C**) Venn diagram depicting the distribution of hits as determined by manual and computational analyses of images acquired during functional genomic screening. (**D**) Comparative gene ontology (GO; biological process) enrichment analysis carried out for manually identified hits (Man), MΦ-Candescence hits (Can), and common hits (Both). Dot size indicates the number of genes associated with a given GO term, and color indicates method by which those genes were identified. GO terms within a cluster share similar biological functions. Enrichment analysis was performed with ViSEAGO using the weight01 algorithm.

We screened the *C. albicans* GRACE (2,323 genes) (24) and GRACEv2 (857 genes) (25) collections to identify mutants that were unable to filament upon internalization by macrophage-like phagocytes in culture (Table S1A). GRACE strains were co-cultured for 4 h with mouse monocyte-macrophage lineage J774A.1 cells (MΦ) in the presence of 5 µg/mL DOX, then stained with fluorescein isothiocyanate (FITC)-conjugated anti-*C*. *albicans* antibody, and imaged to visualize fungal cell morphology (Fig. 1A). We used two approaches to identify mutants that were impaired in their ability to filament in phagocytes. Images of GRACE mutants cultured with phagocytes were first scored manually on a scale of 0 to 2, where 0 represents yeast, 1 represents short filaments, and 2 represents filaments with a length comparable to wild type (Fig. 1A). Images in which there were too few fungal cells to identify a phenotype were labeled as having a growth defect (GD). For the manual method, mutants blocked in intraphagosomal filamentation (hits) were considered to be those that scored 0 or 1 in co-culture with phagocytes in both biological replicates (Fig. 1A; Table S1B). Microscopy images were also analyzed using Candescence, a deep learning-based approach that automatically recognizes *C. albicans* cells and labels them according to their morphology (26). Using transfer learning to extend the neural network underlying the original Candescence program to our image compendium, we developed a classifier for labeling *C. albicans* cells in co-culture with phagocytes (MΦ-Candescence, Text S1, and Fig. S1). MΦ-Candescence used a labeling system similar to the manual method (Fig. S1A), but individually labeled every fungal cell in an image (Fig. S1B), in contrast to the manual method that assigned each image an overall score. MΦ-Candescence included an additional label of 3 for phagocytes that had internalized multiple *C. albicans* cells, but for which individual filaments could not be resolved (Fig. S1A). To detect hits using MΦ-Candescence, we determined the average fraction of non-filamentous cells for each strain in phagocytes by calculating the proportion of internalized *C. albicans* cells labeled as non-filamentous (0 or 1) relative to the total number of internalized fungal cells, averaged across the two replicates. GRACE strains with a minimum average of 30 fungal cells per image, corresponding to the 95th percentile of strains labeled manually as GD, and with an average fraction of non-filamentous cells exceeding 70.5% were considered hits (Fig. 1B).

Using both manual scoring and MΦ-Candescence, we identified 359 genes important for *C. albicans* filamentation inside phagocytes (Fig. 1C; Table S1B). 85 of 359 genes were identified by both analyses, which is a significant overlap (hypergeometric test, *P* < 10^−10^). The manual method identified an additional 108 genes as required for filamentation in phagocytes, and MΦ-Candescence identified an additional 166 genes (Fig. 1C). Differences in the genes identified by each mode of analysis are likely due to the inherent methodology differences between manual annotation, which assigns an overall qualitative score to each image, and annotation by MΦ-Candescence, which quantifies the fraction of non-filamentous cells. MΦ-Candescence appeared better able to identify hits from densely populated images, whereas the manual method identified 30 genes with an average of less than 30 fungal cells per image that were excluded by MΦ-Candescence despite containing a sufficient number of fungal cells with a consistent filamentation phenotype to enable a manual score to be assigned. With different benefits and weaknesses inherent to each method, we elected to consider results from both in moving the study forward.

Comparative gene ontology (GO; biological process) enrichment analysis was carried out for manually identified hits, MΦ-Candescence hits, and common hits (Fig. 1D; Table S1C). Genes identified by the manual method were significantly enriched (*P* < 0.01) in the greatest number of biological processes, including those previously implicated in filamentation, such as the ergosterol biosynthesis pathway (27) and Arp2/3 complex-mediated actin nucleation (28) (Fig. 1D). Additionally, GO terms associated with filamentous growth (cluster 6) were significantly enriched, validating our screening approach (Fig. 1D). Genes involved in ATP synthesis-coupled proton transport, translation, and protein import into the mitochondrial matrix were significantly enriched within the gene set identified by both methods. Additionally, MΦ-Candescence identified an enrichment for genes involved in the cellular response to unfolded proteins, which was not captured by the manual annotation.

**FIG 2:**
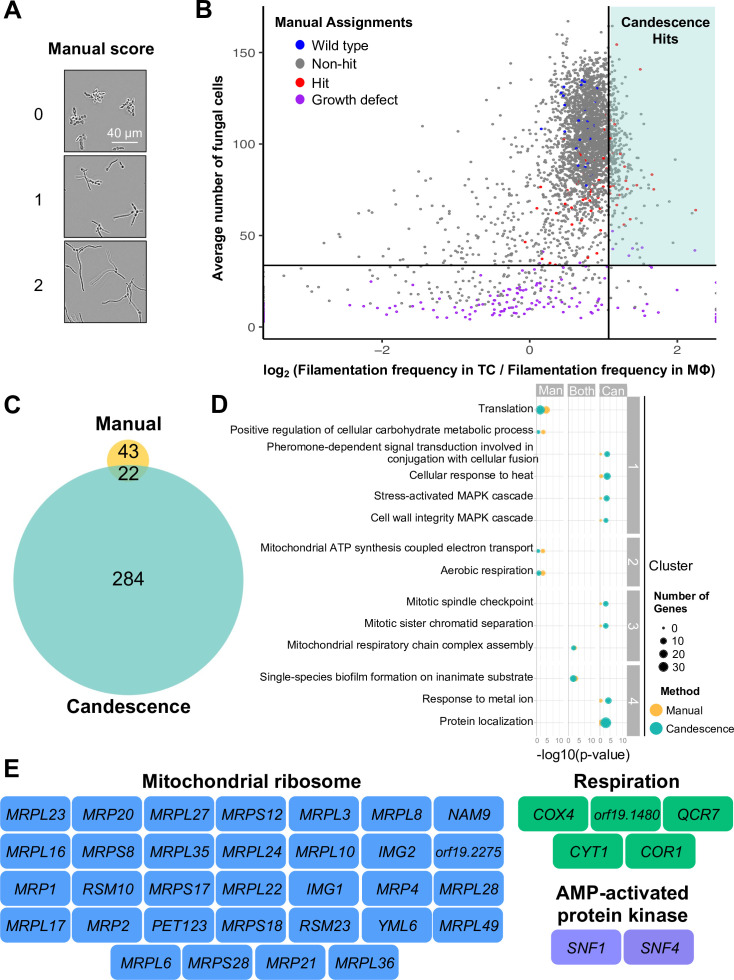
Filamentation in phagocytes displays a distinct dependence on respiration-based alternative carbon source utilization. (**A**) The degree of filamentation for each GRACE strain was manually scored after monoculture without phagocytes under standard TC conditions. GRACE strains were grown overnight in the presence of low DOX prior to culture under standard TC conditions for 4 h in the presence of high DOX to maximally repress gene expression. Cultures were then fixed and imaged to enable the degree of filamentation to be scored for each strain. Representative images of a strain from each manual scoring category are shown. (**B**) The log_2_ ratio of the frequency of filamentous cells in monoculture TC images determined by TC-Candescence divided by the frequency of filamentous cells in phagocyte (MΦ) co-culture images determined by MΦ-Candescence for each GRACE strain (*x*-axis) is plotted versus the geometric average of the number of fungal cells detected by Candescence algorithms in each image (*y*-axis). The vertical line marks the 90th quantile, with positive Candescence predictions defined as those strains lying to the right of this line. The horizontal line marks the 95th quantile of all strains manually labeled as having a growth defect (purple dots). Blue dots correspond to wild-type control strains present on multiple GRACE library plates; red dots correspond to strains identified as hits by manual analysis; and gray dots correspond to all other strains. The turquoise-shaded quadrant indicates strains selectively impaired in their ability to filament in MΦ relative to monoculture under TC conditions. (**C**) Venn diagram depicting the distribution of hits as determined by manual and computational annotation of images acquired during functional genomic screening. (D) Comparative GO enrichment analysis carried out for manual hits (Man), Candescence hits (Can), and common hits (Both). Dot size indicates the number of genes associated with a given GO term, and color indicates the method by which those genes were identified. GO terms within a cluster share similar biological functions. Enrichment analysis was performed with ViSEAGO using the weight01 algorithm. (**E**) Functional classification of genes required for filamentation in phagocytes but not in monoculture highlights dependence on respiration-based alternative carbon source utilization. Genes identified manually and/or by Candescence that encode subunits of the mitochondrial ribosome, electron transport chain, and AMPK complex are indicated in boxes color-coded by category.

Given that the TC conditions (RPMI, 3% serum, 37°C, 5% CO_2_) we used to support phagocyte growth induce *C. albicans* filamentation, we also screened the GRACE and GRACEv2 collections in the absence of phagocytes, enabling us to differentiate genes that are required for intraphagosomal filamentation from those required under TC conditions in the absence of phagocytes. Akin to our co-culture screen, we assessed the ability of each mutant to filament under TC conditions manually and using a second novel Candescence-based classifier (TC-Candescence). The manual approach assigned each image a score ranging from 0 to 2 based on the filamentation phenotype of each strain in TC conditions (Fig. 2A). Mutants selectively blocked in intraphagosomal filamentation (hits) were considered those that scored 0 or 1 in co-culture with phagocytes but 2 in the absence of phagocytes in both biological replicates of each screen. TC-Candescence used a similar scoring approach, but with a range from 0 to 3, where 3 was used to label stellate clusters of filamentous cells (Text S1, Fig. S2A). To assign genes as hits using Candescence, we determined the log-ratio of filamentation frequency in TC conditions versus the filamentation frequency in phagocytes for each strain. For each condition, filamentation frequency was calculated by dividing the number of *C. albicans* cells per image, which scored 2 or 3, by the total number of fungal cells in the image and then averaging the values obtained for each replicate. Strains with a log-ratio greater than 0 were those with a greater proportion of filamentous cells in the absence of phagocytes. GRACE strains with a minimum average of 33.7 fungal cells per image, which corresponds to the 95th percentile of strains labeled manually as GD, and with a log-ratio of frequencies that fell beyond the 90% quantile were considered hits (Fig. 2B).

Manual scoring identified 65 genes that were required for *C. albicans* filamentation in phagocytes, but not under TC conditions in the absence of phagocytes. Candescence identified 306 genes, and 22 of these were also identified by the manual method, which is a significant overlap (hypergeometric test, *P* < 10^−7^, Fig. 2C; Table S1D). Comparative GO enrichment analysis of the genes selectively required for intraphagosomal filamentation revealed 14 significantly enriched biological processes (Fig. 2D; Table S1E). Genes involved in mitochondrial respiratory chain complex assembly and single-species biofilm formation on inanimate substrates were enriched when considering analyses from both methods (Fig. 2D; Table S1E). Translation, carbohydrate metabolism, and aerobic respiration were identified by the manual method alone, whereas pheromone, heat, and mitogen-activated protein kinase signaling; mitosis; metal ion response; and protein localization were identified by Candescence alone (Fig. 2D; Table S1E).

Further investigation of the genes underlying each biological process found that 8 of the 14 genes identified as hits from the manual scoring method that are involved in translation encode subunits of the mitochondrial ribosome (Table S1E). Candescence identified 24 additional genes annotated as encoding mitochondrial ribosome subunits (Fig. 2E). Notably, the mitochondrial ribosome is required for translation of the 14 mRNAs encoded by the *C. albicans* mitochondrial genome, all of which encode subunits of complexes in the electron transport chain (ETC) (29). We also identified five nuclear genes that encode ETC complex subunits (Fig. 2E). Additionally, we identified two out of three subunits of the AMP-activated SNF1-based kinase complex, which is involved in upregulating the expression of genes needed for alternative carbon source utilization through pathways that converge on respiration to generate energy (Fig. 2E) (30, 31). Together, these data suggest that *C. albicans* relies on respiration to support intraphagosomal filamentation.

### Compromise of respiration in the absence of glucose impairs *C. albicans* filamentation and depletes ATP

Based on the GO-enrichment signatures obtained from our primary screens, we subsequently focused on the reliance of *C. albicans* on respiration during its interaction with phagocytes. We chose three representative mutants to characterize this phenotype: *MRP21*, which encodes a subunit of the mitochondrial ribosome; *COR1*, which encodes a subunit of ETC complex III; and *SNF1*, which encodes the catalytic subunit of the sole AMPK in *C. albicans*. We confirmed DOX-dependent repression of the target gene in each GRACE strain (Fig. S3A), providing confidence that phenotypic changes in these strains in the presence of DOX were due to repression of the target gene. Additionally, given that cell viability is a prerequisite for filamentation, we confirmed that all mutants remained viable both prior to and after 4 h of co-culture with phagocytes in the presence of DOX (Fig. S3B).

**FIG 3:**
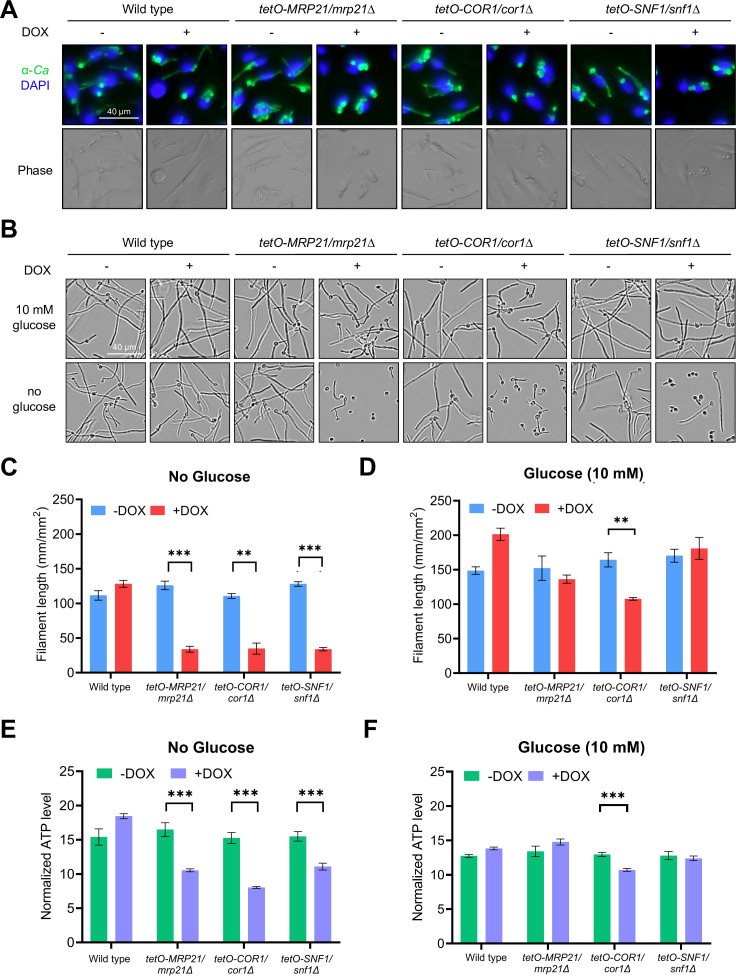
Compromise of respiration in the absence of glucose impairs filamentation and depletes ATP levels. (**A**) Expression of representative genes encoding subunits of the mitochondrial ribosome (*MRP21*), electron transport chain (*COR1*), or AMPK complex (*SNF1*), which are required for *C. albicans* filamentation in J774A.1 cells, are also required for filamentation in BMDMs. GRACE strains were grown overnight in the absence or presence of low DOX prior to co-culture with BMDMs for 4 h in the absence or presence of high DOX to maximally repress gene expression. Co-cultures were then fixed, permeabilized, and stained with FITC-conjugated anti-*C*. *albicans* antibody (α*Ca*, green signal). DAPI was used to stain phagocyte nuclei (blue signal). (**B**) *MRP21*, *COR1*, and *SNF1* are needed for filamentation in the absence of glucose. GRACE strains and the corresponding wild-type control were grown overnight in the presence or absence of low DOX prior to culture for 4 h in glucose-free RPMI medium without or with supplementation with glucose (10 mM) and in the absence or presence of high DOX. (**C and D**) Quantification of filament length (mm/mm^2^) in images shown in panel **B** for strains in the absence (**C**) or presence (**D**) of glucose as measured using the IncuCyte NeuroTrack Processing Module. (**E and F**) *MRP21*, *COR1*, and *SNF1* are important in the absence of glucose (**E**) but dispensable in the presence of glucose (**F**) for maintaining ATP levels. GRACE strains were grown overnight in the presence or absence of low DOX, subcultured, and grown for 3 h with or without high DOX, and cultured in glucose-free RPMI medium without or with supplementation with glucose (10 mM) in the absence or presence of high DOX. After an hour, ATP levels were measured by a luciferase-based assay and normalized by relative cell number in the well (OD_600_). Data are representative of two biological replicates (*n* = 2). For panels **C–F**, error bars indicate the standard deviation for technical triplicates, and statistical significance was calculated using a two-sided unpaired *t*-test. ^∗∗^*P* ≤ 0.01; ^∗∗∗^*P* ≤ 0.001. Significance is reported for strains demonstrating a significant decrease in filament length or ATP level in the presence of DOX.

We next examined the filamentation phenotype of each strain after engulfment by mouse bone marrow-derived macrophages (BMDMs) to determine if the block in intraphagosomal filamentation observed in these strains also occurs in primary macrophages, which do not exhibit the constitutive aerobic glycolysis (Warburg effect) typical of J774A.1 and many other cancer cell lines (32–34). Instead, these primary cells shift to glycolysis only upon activation by co-culture with *C. albicans* and other microbial pathogens (35). All mutants remained blocked in filamentation in BMDMs in a DOX-dependent manner after 4 h of co-culture (Fig. 3A).

A feature that distinguishes the phagosome environment from the TC medium is the availability of glucose. Glucose is present at ~11 mM in TC medium, where *C. albicans* undergoes filamentation even when genes encoding components of the mitochondrial ribosome, ETC, or AMPK are repressed, while minimal glucose is thought to be available within the phagosome (17, 36). Therefore, we hypothesized that upon repression of genes encoding components of the mitochondrial ribosome, ETC, or AMPK, *C. albicans* would be unable to filament in glucose-free RPMI, where amino acids are present as the major carbon source. Indeed, mutants were unable to filament after incubation for 4 h at 37°C and 5% CO_2_ in glucose- and serum-free RPMI with DOX (Fig. 3B and C; Fig. S4A). Notably, these mutants retained their ability to filament in serum-free RPMI medium containing glucose and DOX (Fig. 3D; Fig. S4B). We observed similar results in RPMI medium adjusted to pH 5.5 to better recapitulate the acidic environment of the phagosome (Fig. S4C and D).

Next, to determine whether fungal ATP content was altered in mitochondrial ribosome, ETC, and AMPK subunit mutants under conditions that impede filamentation, we measured ATP levels in glucose- and serum-free RPMI medium. As a positive control, ATP content was examined in a strain lacking *RIP1*. In this deletion strain, ATP should be reduced upon growth in media lacking glucose. Like *COR1*, *RIP1* encodes a subunit of ETC complex III but is not present in the GRACE libraries we screened and was therefore not previously identified. Indeed, in a *rip1*Δ/Δ mutant, we observed a significant decrease in ATP in RPMI medium formulated without glucose, which correlated with reduced filamentation (Fig. S5). Likewise, we observed a significant reduction in ATP levels in the *MRP21*, *COR1*, and *SNF1* GRACE strains when cultured in glucose-free RPMI medium containing DOX, relative to the absence of DOX (Fig. 3E; Fig. S5D and E). Notably, we observed little to no DOX-dependent differences in ATP content in these strains in glucose-free RPMI to which 10 mM glucose was added back (Fig. 3F; Fig. S5F and G). Together, results suggest that *C. albicans* relies on the mitochondrial ribosome, ETC, and AMPK to generate ATP and filament under culture conditions where only non-fermentable carbon sources are available.

### *C. albicans* intraphagosomal filamentation is dependent on extracellular glucose and the glycolytic metabolism of phagocytes

The reliance of *C. albicans* on mitochondrial respiration for intraphagosomal filamentation is consistent with literature indicating that the phagosome is devoid of fermentable carbon sources, such as glucose, from which *C. albicans* can generate energy via glycolysis (17). However, non-fermentable carbon sources could still originate from the metabolism of glucose by the host phagocyte. To investigate further, we examined the role of extracellular glucose in the culture medium as a potential nutrient source capable of enabling *C. albicans* filamentation within the phagosome. For these experiments, we used a different phagocytic mouse cell line of macrophage-monocyte lineage, RAW264.7. Confirming the generalizability of our findings, *MRP21*, *COR1*, and *SNF1* GRACE mutants were also blocked in filamentation in RAW264.7 cells 4 h post-infection in the presence of DOX (Fig. S3C).

**FIG 4:**
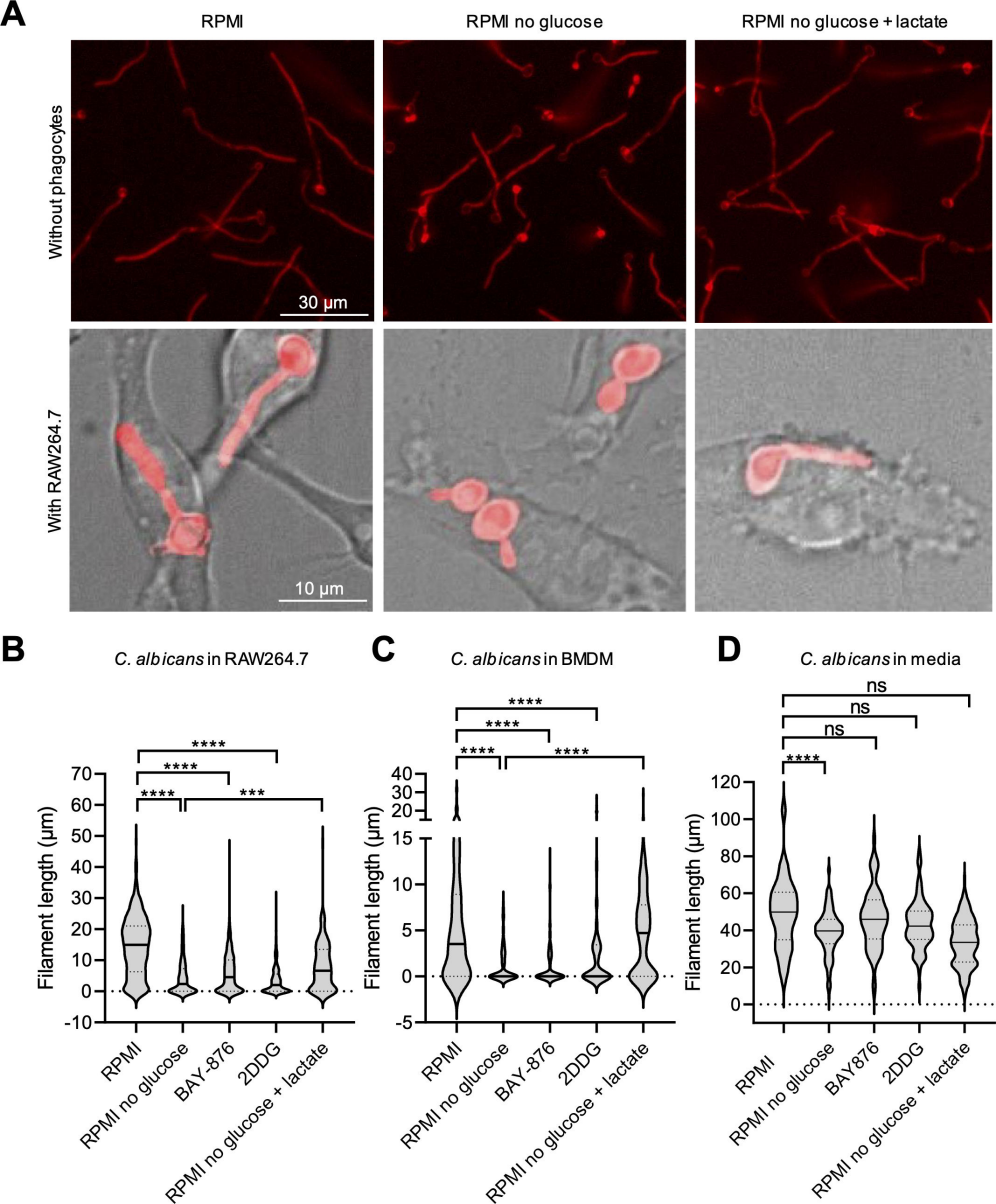
Intraphagosomal filamentation of *C. albicans* is dependent on phagocyte glycolysis. (**A**) Representative images of *C. albicans* grown for 3 h in the absence (top panel) or presence (bottom panel) of RAW264.7 cells in RPMI, RPMI without glucose, or RPMI without glucose + lactate (15 mM). (**B and C**) Measurement of intraphagosomal filament length of *C. albicans* in RAW264.7 cells 3 h post-infection (**B**) or in BMDMs 4 h post-infection (**C**). After co-incubation of fungi with phagocytes for 45 minutes, co-cultures were washed and medium replaced with: RPMI, RPMI without glucose, RPMI with BAY-876 (5 µM), RPMI with 2-deoxy-D-glucose (10 mM), or RPMI without glucose + lactate (15 mM). (**D**) *C. albicans* growth after 3 h under monoculture conditions in media as described in panels **B** and **C**. For panels **B–D**, data are presented as violin plots, where the middle solid line indicates the median and the lower and upper dashed lines indicate the first and third quartiles, respectively. Data were analyzed by one-way analysis of variance (Tukey’s multiple comparisons test) to compare the means of five groups. ns (non significant) = *P* > 0.05, ^∗∗∗^*P* ≤ 0.001, ^∗∗∗∗^*P* ≤ 0.0001. The data are representative of three biological replicates (*n* = 3).

Having validated the system, we next examined the impact of depleting glucose from the culture medium during co-culture of *C. albicans* with RAW264.7 cells. Glucose was removed prior to infection with *C. albicans* to avoid trapping of the sugar in nascent phagosomes and then either added back 45 minutes post-infection or omitted for the remainder of the experiment, as indicated. This approach ensured that the extent of phagocytosis would be comparable under both conditions. Serum was omitted from both the glucose-containing and glucose-deficient media. Interestingly, relative to standard glucose-supplemented RPMI (~11 mM glucose), co-culture in glucose-free RPMI (RPMI without glucose) led to a marked reduction in the intraphagosomal filament length of *C. albicans* (Fig. 4A and B). Similar to RAW264.7 cells, co-culture of BMDMs in glucose-free RPMI led to a significant reduction in the intraphagosomal filament length of *C. albicans* (Fig. 4C). While incubating *C. albicans* in glucose-free RPMI in the absence of phagocytes also led to a modest decrease in filament length (~20% reduction, Fig. 4D), the reduction was less than that observed in the presence of phagocytes (~80% reduction, Fig. 4B). Taken together, results indicate that intraphagosomal filamentation of *C. albicans* is dependent on the presence of millimolar concentrations of extracellular glucose in the culture medium.

To further probe the reliance of fungal filamentation in the phagosome on the uptake of extracellular glucose by phagocytes, we supplemented culture medium with BAY-876, an inhibitor of the glucose transporter GLUT1 (37). Supplementation of standard glucose-containing RPMI with BAY-876 reduced the intraphagosomal filament length of *C. albicans* in both RAW264.7 cells and BMDMs (Fig. 4B and C), but it did not impact *C. albicans* filamentation in the absence of phagocytes (Fig. 4D). Notably, BAY-876 also had little to no impact on *C. albicans* filamentation or growth in glucose-free RPMI medium adjusted to either pH 7 or pH 5.5 (Fig. S6A and B) and did not reduce macrophage viability (Fig. S7A). Together, these data suggest that intraphagosomal filamentation is dependent on the transport of extracellular glucose into phagocytes via GLUT1.

Given previous literature (17) and our supporting findings that *C. albicans* relies on non-fermentable carbon sources to generate energy via respiration for filamentation in phagocytes (Fig. 2E), we reasoned that a metabolite of glucose generated within RAW264.7 cells during glycolysis, rather than glucose itself, is most likely utilized as a nutrient source by *C. albicans* in the phagosome. After uptake of glucose by GLUT1, glucose is rapidly phosphorylated by hexokinase in the cytosol to form glucose-6-phosphate, the first step of glycolysis (38). The glucose analog 2-deoxy-D-glucose (2DDG) can likewise be phosphorylated but cannot undergo isomerization by phosphoglucose isomerase, the second step of glycolysis, thereby competitively inhibiting glycolysis from proceeding to generate glucose-derived metabolites (38, 39). We found that adding 2DDG to the culture medium resulted in a decrease in the intraphagosomal filament length of *C. albicans* in both RAW264.7 cells and BMDMs (Fig. 4B and C) but did not reduce macrophage viability (Fig. S7A) or filamentation in the absence of phagocytes (Fig. 4D). While 2DDG had no impact on *C. albicans* filamentation in RPMI under neutral or acidic pH, it resulted in a significant decrease in *C. albicans* growth (Fig. S6C and D), suggesting that the impact of 2DDG on *C. albicans* growth and filamentation is uncoupled. We did not examine the impact of 2DDG on *C. albicans* growth and filamentation in glucose-free RPMI because glycolytic flux is already shut down in the absence of glucose, and any impact of 2DDG on *C. albicans* under these conditions would likely be due to non-glycolysis-related effects (40). While the lack of effect of 2DDG on *C. albicans* filamentation in monoculture could be due to ineffective exposure to the compound, the growth inhibition 2DDG caused argues against this possibility. Taken together with the ability of *C. albicans* to filament in glucose-free medium, these results support the overall conclusion that fungal glycolysis is not required for intraphagosomal filamentation. Altogether, these data suggest a model in which limiting glycolysis in phagocytes diminishes the availability of alternative carbon sources within the phagosome, impairing *C. albicans* filamentation.

Metabolism of glucose by mammalian cells via glycolysis results in the production of pyruvate or lactate, depending on the presence of oxygen and the energy state of the cell (38). The switch from respiration to aerobic glycolysis that occurs in innate immune cells such as macrophages after phagocytosis of a pathogen results in elevated production of lactate (35). Therefore, we reasoned that glucose-derived lactate might be utilized by *C. albicans* as an alternative carbon source to enable filamentation within the macrophage phagosome. Interestingly, we found that the addition of lactate partially rescued the decrease in *C. albicans* filamentation within RAW264.7 cells and BMDMs observed during co-culture in glucose-free RPMI (Fig. 4A, B and C). Notably, supplementation with lactate did not result in a significant increase in filamentation of *C. albicans* grown in the absence of phagocytes, relative to growth in glucose-free RPMI (Fig. 4A and D). Jointly, these data suggest that lactate in the extracellular environment can compensate for a lack of extracellular glucose to enable *C. albicans* filamentation in macrophages.

Lactate is transported across cytoplasmic and intracellular membranes by several monocarboxylate transporters (MCTs), of which SLC16A1 (MCT1) and SLC16A3 (MCT4) are present in the phagosomes and lysosomes of macrophages (41, 42). Therefore, we investigated the impact of inhibiting monocarboxylate transport with BAY-8002 and Bindarit, which have been reported to inhibit SLC16A1 and SLC16A3, respectively (43, 44). Incubation of co-cultures in glucose-containing RPMI supplemented with Bindarit but not BAY-8002 resulted in a significant reduction in *C. albicans* intraphagosomal filament length (Fig. S7B). Notably, when *C. albicans* was cultured with Bindarit in the absence of phagocytes, we did not observe any differences in filament length (Fig. S7C). To gain further insight, we generated RAW264.7 cell lines in which SLC16A3 was knocked out (KO5, KO10, KO11) or knocked down via RNA interference (siRNA). Despite confirming reduced SLC16A3 transcript levels in these cell lines (Fig. S7D), we did not observe a significant decrease in the intraphagosomal filament length of *C. albicans* during co-culture in standard RPMI medium (Fig. S7E). Together, these data suggest that the observed effects of Bindarit were due to the off-target activity of this compound and that phagocyte SLC16A3 is not essential for intraphagosomal filamentation of *C. albicans*. Lactate may be delivered across the phagosomal membrane by transporter(s) other than SLC16A1 or SLC16A3 or may be converted into another metabolite that can traverse the phagosomal membrane and similarly serve as a non-fermentable carbon source to support filamentation.

### *C. albicans* relies on respiration for intraphagosomal growth, phagosome escape, and phagocyte killing

**FIG 5:**
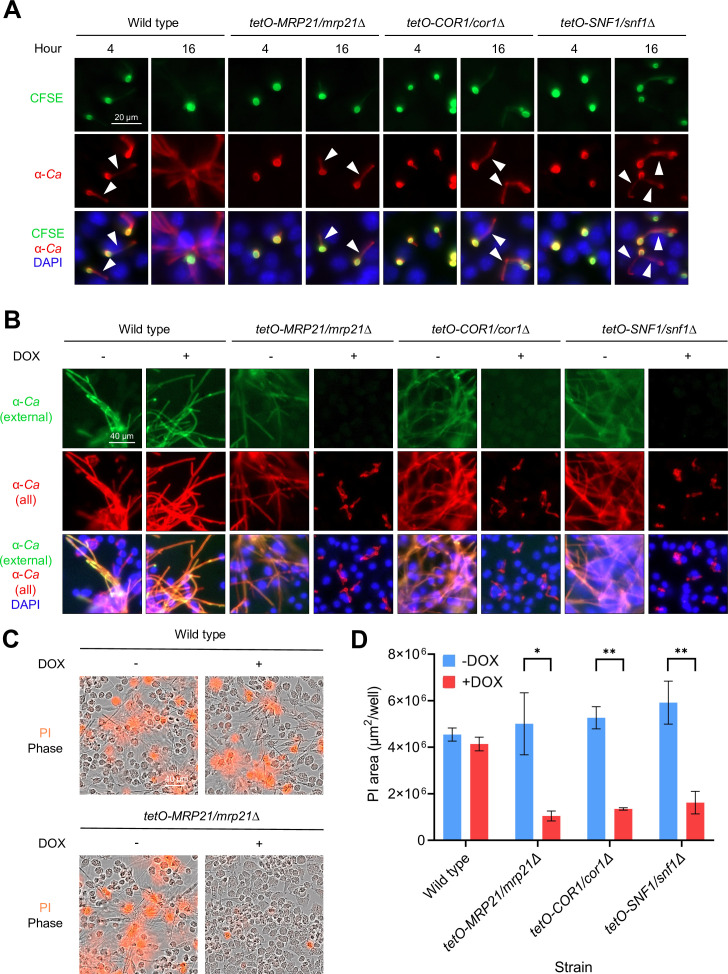
*C. albicans* requires respiration to escape from and kill macrophages. (**A**) Expression of representative genes encoding subunits of the mitochondrial ribosome (*MRP21*), electron transport chain (*COR1*), or AMPK complex (*SNF1*) enables intraphagosomal growth. GRACE strains were grown overnight in the presence of low DOX and stained with CFSE (green signal) to label mother cells prior to co-culture with J774A.1 cells in the presence of high DOX to maximally repress gene expression. Co-cultures were fixed at 4 or 16 h, permeabilized, and incubated with anti-*C*. *albicans* antibody and AF555-conjugated secondary antibody to label all *C. albicans* cells (red signal). DAPI was used to stain phagocyte nuclei (blue signal). (**B**) *MRP21*, *COR1*, and *SNF1* are required for *C. albicans* to escape macrophages. GRACE strains were grown overnight in the absence or presence of low DOX prior to co-culture with J774A.1 cells in the absence or presence of high DOX. Co-cultures were fixed at 16 h and then incubated with FITC-conjugated anti-*C*. *albicans* antibody (α*Ca,* green signal) to stain only external *C. albicans* cells. Antibody was washed away, and co-cultures were then permeabilized prior to incubation with anti-*C*. *albicans* antibody and AF555-conjugated secondary antibody to label all *C. albicans* cells (red signal). DAPI was used to stain phagocyte nuclei (blue signal). (**C and D**) *MRP21*, *COR1*, and *SNF1* are required for *C. albicans* to kill phagocytes. GRACE strains were grown overnight in the absence or presence of low DOX prior to co-culture with J774A.1 cells for 16 h in the absence or presence of high DOX. Propidium iodide was included to label lysed phagocytes (red signal). (**C**) Representative images of co-cultures of J774A.1 cells with wild-type and *tetO-MRP21/mrp21∆ C. albicans* strains.(**D**) Quantification of phagocyte killing by wild-type and mutant *C. albicans* strains in the absence or presence of DOX. For panel **D**, error bars indicate standard deviation for technical triplicates, and statistical significance was calculated using a two-sided unpaired *t*-test. ^∗^*P* ≤ 0.05; ^∗∗^*P* ≤ 0.01. The data are representative of two biological replicates (*n* = 2).

While inhibition of glycolysis in phagocytes and repression of *C. albicans* genes encoding components of the mitochondrial ribosome, ETC, and AMPK resulted in a decrease in intraphagosomal filamentation at early time points (4 h), we wondered about the phenotype of these mutants at later time points in the *C. albicans*-macrophage interaction. To better resolve filaments formed at late time points (Phase II of the post-infection period), we stained mutants with carboxyfluorescein succinimidyl ester (CFSE; green), which brightly labels the cell wall of the mother cell but is poorly transferred to the emergent germ tube. CFSE-labeled *C. albicans* cells were co-cultured with phagocytes for 4 or 16 h. Co-cultures were then fixed, permeabilized, and incubated with anti-*C*. *albicans* primary antibody and AF555-conjugated secondary antibody (red) to label both mother cells and germ tubes residing within and outside the phagocytes. As we found previously, the mutants remained blocked in filamentation at 4 h in the presence of DOX (Fig. 5A). However, at 16 h, the mutants formed short filaments similar in length to those formed by wild-type *C. albicans* at 4 h (Fig. 5A, white arrows), suggesting that filamentation is severely delayed but not completely blocked in these mutants. Although *C. albicans* respiration has been previously linked with filamentation through signaling pathways that are independent of growth (45), the delay in filamentation we observed suggests that genes encoding components of the mitochondrial ribosome, ETC, and AMPK are important for energy production to support expansion of *C. albicans* biomass within the phagosome environment, where glucose is limited, rather than strictly filamentation *per se*. Consistent with these conclusions, we observed a DOX-dependent decrease in the growth of these mutants when cultured for 24 h at 30°C in glucose-free RPMI medium adjusted to pH 7 or pH 5.5 (Fig. S4EF).

To characterize the impact of the growth defect of these mutants in phagocytes on outcomes of the *C. albicans*-phagocyte interaction, we examined the ability of the mutants to escape from and kill phagocytes (Phase II death). To distinguish between escaped and internally retained *C. albicans*, we fixed co-cultures at 16 h post-inoculation and incubated them with FITC-conjugated anti-*C*. *albicans* antibody (green), thereby staining only extracellular, escaped *C. albicans* cells. Subsequently, we permeabilized the co-cultures and incubated them with anti-*C*. *albicans* primary antibody and AF555-conjugated secondary antibody (red), thereby staining all *C. albicans* cells, both internally retained and escaped. Consistent with previous reports (11), wild-type *C. albicans* formed robust filaments and escaped; however, in the presence, but not absence, of DOX, the mutants remained internalized within phagocytes, as evidenced by a lack of green staining (Fig. 5B). We next examined the ability of the mutants to kill J774A.1 cells using propidium iodide (PI) staining to identify dead phagocytes. After co-culture for 16 h, we used imaging to identify dead phagocytes, which were unable to exclude PI, and visualize the morphology of the *C. albicans* strains. Representative images obtained with wild-type and *tetO-MRP21/mrp21∆* strains demonstrate that wild-type *C. albicans* induced pronounced phagocyte death in the absence and presence of DOX, while DOX treatment resulted in a major impairment in phagocyte death only with the mutant strain (Fig. 5C). By quantifying the extent of mammalian cell death, we found that down-regulating expression of each target gene caused a significant reduction in phagocyte cell death (Fig. 5D; Fig. S8). We conclude that respiration and AMPK activity play key roles in enabling *C. albicans* to kill and escape from phagocytes.

## DISCUSSION

Filamentation and metabolic flexibility are key virulence traits of *C. albicans* that enable disease in the host (46–48). Here, we employed functional genomic screening to identify genes selectively required for the growth and filamentation of this pathogen in macrophages. Through manual and machine learning image analyses, we converged on respiration and AMPK function as key effectors of alternative carbon metabolism needed by *C. albicans* to expand within the phagosome, escape, and induce phagocyte cell death. Furthermore, we found that intraphagosomal filamentation of *C. albicans* is dependent on glucose uptake and its subsequent glycolytic metabolism by phagocytes, thereby identifying a role for extracellular glucose during Phase I of the *C. albicans*-phagocyte interaction.

As it matures, the phagosome presents an increasingly hostile environment where nutrients become limited and pathogens are confronted by an arsenal of antimicrobial forces (49). Nutrient deprivation, oxidative stress, altered pH, and even phagocyte-derived proteins are known to induce *C. albicans* filamentation *in vitro* (50, 51), but the entire repertoire of triggers contributing to the induction of *C. albicans* filamentation in macrophages has yet to be defined (52). Our data reveal extensive overlap in the genes required for filamentation in phagocytes and those that enable filamentation in monoculture under tissue culture conditions, which suggests that shared factors act to stimulate filamentation in both environments. However, we also identified over 300 genes that, when depleted, had a greater impact on filamentation in phagocytes than in monoculture, demonstrating that distinct genetic circuitry enables intraphagosomal filamentation of *C. albicans*. While we found *C. albicans* respiration to be required for biomass expansion rather than strictly for filamentation within the phagosome, several others have implicated *C. albicans* respiration in filamentation (45, 53, 54). Thus, the mechanisms by which respiration integrates diverse extracellular and intracellular signals to impact *C. albicans* morphology is complex and extends beyond its role in ATP production to enable growth.

Our screen of the GRACE library in co-culture with phagocytes (Fig. 1) identified known positive regulators of morphogenesis, such as *CDC42* and *FLO8*, validating our approach. However, there may have been instances where the DOX concentration used (5 µg/mL) was insufficient to achieve repression of the target gene to the point of generating an observable phenotype. Such a possibility could explain why other known morphogenetic regulators, such as *RAS1* and *CYR1*, were not identified. Additionally, even when target gene levels are sufficiently repressed, protein half-life may be long, such that functional gene product remains over the course of the assay. Indeed, this may be the case for *MRP21* and *COR1*, whose orthologs in *Saccharomyces cerevisiae* are reported to have protein half-lives of 4.6 and 8.9 h, respectively (55). While we confirmed transcriptional repression of *MRP21* and *COR1* in the corresponding GRACE mutants used in our study (Fig. S3A), we appreciate that the protein product of these genes may have lingered in the cell. The presence of these proteins could explain why *tetO-MRP21/mrp21*Δ and *tetO-COR1/cor1*Δ mutants remained viable after treatment with DOX, despite evidence suggesting that these genes may be essential in *C. albicans* (56). Finally, an additional limitation of this work is the fact that the GRACE and GRACEv2 collections still do not achieve genome-scale coverage, with the result that some genes, such as *EFG1*, were not identified as regulators of filamentation simply because they are not present in the library. Our ongoing expansion of this resource will undoubtedly uncover additional genes with important roles in filamentation.

The shift to aerobic glycolysis that occurs in phagocytes upon sensing microbial ligands enhances the generation of reactive oxygen species while still providing enough energy to support antimicrobial inflammation and the production of cytokines important for activation of host defenses (57–60). However, this metabolic rewiring comes at a cost, as macrophages become dependent on glucose for survival (14, 61). *C. albicans* exploits this liability after it escapes from the phagosome by rapidly consuming extracellular glucose and triggering macrophage cell death (Phase II) (14). Our study suggests an additional role for glucose early in the *C. albicans*-phagocyte interaction (Phase I), where we found that limiting glucose availability, uptake, or glycolysis impairs *C. albicans* filamentation. Therefore, the Warburg shift in macrophages not only contributes to their eventual Phase II death but is also exploited to provide alternative carbon sources to support fungal respiration and enable intraphagosomal filamentation of *C. albicans,* which is tied to Phase I death and fungal escape. In parallel, the metabolic shift by *C. albicans* to respiration upon internalization by phagocytes provides a mechanism by which the fungus can both expand within the glucose-limited phagosome and avoid destruction.

Given the limited antifungal armamentarium, there is an urgent need to develop new therapeutic strategies to combat fungal infections (62). Targeting pathogen virulence traits is an alternative to classical antimicrobial therapies, which inhibit growth or kill the pathogen and are prone to the development of antimicrobial resistance (63). Anti-virulence therapies target the mechanisms that pathogens use to cause disease, disarming organisms without affecting physiological commensal relationships (63). While some anti-virulence therapies against pathogenic bacteria have been approved for clinical use, targeting virulence remains an underexplored strategy for treating fungal infections (64, 65). Previous studies have documented the importance of metabolic flexibility in enabling the virulence of *C. albicans* in cell culture models and in mice (23, 66, 67). However, given this flexibility, impairment of multiple alternative carbon metabolic pathways is needed to cause substantial attenuation (19–23). Our study found that impairing respiration or AMPK function is sufficient to compromise the metabolic flexibility of *C. albicans* and cripple its ability to filament in phagocytes, escape, and induce immune cell death. Thus, in contrast to targeting individual metabolic pathways, inhibiting central control nodes such as mitochondrial ETC or AMPK to prevent metabolic adaptation may prove more fruitful as an anti-virulence strategy. In line with this view, studies have shown that genetic inhibition of fungal respiration curtails virulence in mice, and strobilurins, which target ETC complex III, are highly effective as agricultural fungicides (66–68). The role of fungal AMPK in virulence has been less well studied; however, numerous bacterial, viral, and parasitic intracellular pathogens are known to interact with mammalian AMPK in host cells to hijack cellular metabolism for their own benefit (69, 70). Accordingly, pharmacological modulation of mammalian AMPK signaling has been broadly explored as a way to restrict pathogen survival and proliferation (69, 71), but, to our knowledge, targeting the orthologous Snf1-containing complex in fungi to impair virulence has yet to be reported. While identifying and developing fungal-selective molecules remains a challenge, structural studies can provide useful insights for improving the selectivity of molecules whose targets are conserved between fungi and their hosts (72). As an example, the antimalarial atovaquone demonstrates that a species-selective inhibitor of the ETC can be developed as a safe and effective antimicrobial (73). Thus, our work supports the notion that fungal metabolic adaptation represents a valuable therapeutic target for further pursuit.

## MATERIALS and METHODS

### *C. albicans* strains and growth conditions

*C. albicans* strains (Table S1G) were grown under standard laboratory conditions at 30°C in yeast extract-peptone-dextrose (YPD) medium (1% yeast extract, 2% peptone, and 2% d-glucose), unless otherwise stated. Strain archives were maintained in 25% glycerol in YPD medium at −80°C. Unless otherwise stated, *C. albicans* GRACE strains were pinned into flat-bottom 96-well microtiter plates (Sarstedt) containing YPD medium (100 µL/well) and grown overnight at 30°C. On the next day, cells were transferred by pinning (~0.5 uL) into YPD medium (100 µL/well) in the absence and presence of 0.05 µg/mL DOX in 96-well plates and incubated at 30°C prior to subsequent growth or filamentation assays. Strains were constructed as described in Text S1.

### Culture of mammalian cell lines

J774A.1 and RAW 264.7 cells were maintained in RPMI 1640 (RPMI) medium supplemented with 10% heat-inactivated fetal bovine serum (HI-FBS) at 37°C under 5.5% CO_2_.

### Functional genomic screen

J774A.1 cells were diluted to 1 × 10^5^ cells/mL in RPMI medium supplemented with 3% HI-FBS. Cell suspension (100 µL/well) was added to 96-well plates and incubated for 18 h at 37°C under 5.5% CO_2_. On the following day, *C. albicans*-saturated overnight cultures were transferred by pinning into 200 µL of RPMI medium supplemented with 3% HI-FBS and 10 µg/mL DOX (Bio Basic) in 96-well plates. This fungal suspension (100 µL/well) was added to the wells of plates previously seeded with J774A.1 cells for the phagocyte co-culture screen, resulting in a multiplicity of infection favoring engulfment of all fungal cells. RPMI medium supplemented with 3% HI-FBS (100 µL/well) was added to plates with fungal suspension for the monoculture screen without phagocytes under standard TC conditions. Mono and co-culture plates were incubated for 4 h at 37°C under 5.5% CO_2_ then fixed with 4% formaldehyde for 15 min. Formaldehyde was removed, and cells were washed three times with phosphate-buffered saline (PBS, Sigma). In plates containing monocultures, medium was replaced with PBS containing 0.02% sodium azide. Co-cultures were permeabilized with 0.1% Triton-X100 for 15 min, washed three times in PBS, blocked for 15 min in 2% bovine serum albumin (BSA, Sigma, A7030), and stained with FITC-conjugated anti-*C*. *albicans* antibody (1:1,000 dilution, Abcam, ab21164) for 1 h at room temperature. The antibody was removed, cells were washed three times in PBS, and medium was replaced with PBS containing 0.02% sodium azide. Mono and co-culture plates were imaged using the IncuCyte S3 Live-Cell Analysis System (Sartorius).

### BMDM infection with *C. albicans*

BMDMs were prepared as described in Text S1. For Fig. 3A, BMDMs were diluted to 4 × 10^5^ cells/mL in RPMI medium supplemented with 10% HI-FBS, 10 ng/mL recombinant mouse macrophage colony stimulating factor (M-CSF, R&D Systems, 416 mL/CF), 100 U/L penicillin-streptomycin (Gibco), and 2 mM L-glutamine (Gibco). Cell suspension (100 µL/well) was added to 96-well plates and incubated for 18 h at 37°C under 5.5% CO_2_. On the following day, *C. albicans* overnight cultures were diluted to 2 × 10^5^ cells/mL in RPMI medium supplemented as described above in the absence and presence of 10 µg/mL of DOX. The prepared fungal suspension (100 µL/well) was then added to previously seeded BMDMs. Co-cultures were incubated for 4 h at 37°C under 5.5% CO_2_, then fixed, permeabilized, and stained as described above, except 300 ng/mL of 4′,6-diamidino-2-phenylindole (DAPI, Roche) was added to PBS supplemented with 0.02% sodium azide to stain phagocyte nuclei. Images were obtained using an AxioVision inverted microscope (Carl Zeiss) using phase contrast optics and white light illumination or an X-cite series 120 light source for fluorescence excitation. For Fig. 4C, BMDM infection with *C. albicans* was performed as described below for infection of RAW264.7 cells.

### RAW264.7 cell infection with *C. albicans*

siRNA-electroporated and SLC16A3 knockout RAW264.7 cells were constructed as described in Text S1. RAW264.7 or BMDMs cells were sparsely plated on glass coverslips inside 12-well tissue culture plates (Corning Inc.) and grown overnight in RPMI supplemented with 10% HI-FBS. On the next day, medium was replaced with glucose-free RPMI, and phagocytes were infected with rabbit anti-*Candida* IgG-opsonized (OriGene, BP1006) *C. albicans* expressing red fluorescent protein (RFP) for 30 min. Co-cultures were then incubated with donkey anti-rabbit Alexa Fluor 488 (1:1,000 dilution, Jackson, 11-545-152) for 15 min to stain non-internalized yeast cells. After 45 min following the start of co-culture, co-cultures were washed and supplemented with RPMI without glucose supplemented with 15 mM lactate or glucose-containing RPMI with the following pharmacological inhibitors : 5 µM BAY-876, 10 mM 2DDG, 100 µM Bindarit, or 100 µM BAY-8002. Co-cultures were incubated for 3–4 h prior to the acquisition of epifluorescence images using an EVOS M5000 Imaging System (Thermo Fisher Scientific) with a 3.2 MP (2,048 × 1,536) CMOS monochrome camera with 3.45 µm pixel resolution, three-position chamber (470/525, 531/593, 585/624 nm) LED light cubes, and phase contrast imaging mode. Images were acquired using a long working distance of 10×/0.3 N.A. air objective (Invitrogen) and analyzed using ImageJ (Fiji 2.1.0/1.53 c). In some cases, macrophage viability was assessed using the LIVE/DEAD Fixable Green Dead Cell Stain Kit (Thermo Fisher Scientific) after *C. albicans* infection. In this case, donkey anti-rabbit Alexa Fluor 647 (1:1,000 dilution, Jackson, 711-605-152) was used to label the non-internalized yeast cells (see above). All extracellular *C. albicans* cells, as identified by the presence of Alexa Fluor 488 or Alexa Fluor 647 staining, were excluded from measurements. Filament length was manually measured using ImageJ. Macrophage viability per field was manually counted in ImageJ and was calculated as 100% − % green nuclei.

### *C. albicans* filamentation in tissue culture medium

*C. albicans* overnight cultures were diluted to an OD_600_ of 0.006 in glucose-free RPMI medium (Gibco, 11879020) adjusted to either pH 7 or pH 5.5. Fungal suspension (50 µL/well) was added to wells of plates containing glucose-free RPMI medium without or with supplementation with 20 mM glucose and 10 µg/mL DOX (50 µL/well), for a final concentration of 10 mM glucose and 5 µg/mL DOX, and incubated for 4 h at 37°C under 5.5% CO_2_ prior to imaging using the IncuCyte S3 Live-Cell Analysis System. Filament length was quantified via image analysis using the IncuCyte NeuroTrack Processing Module using optimal processing parameters as described previously (74).

### *C. albicans* growth in tissue culture medium

YPD (5 mL) was inoculated with *C. albicans* GRACE strains and grown overnight at 30°C, shaking. On the next day, cells were subcultured to an OD_600_ of 0.1 in YPD (5 mL) in the absence and presence of 0.05 µg/mL DOX and grown overnight at 30°C, shaking. Overnight cultures were diluted to an OD_600_ of 0.1 in glucose-free RPMI medium adjusted to either pH 7 or pH 5.5. Fungal suspension (50 µL/well) was added to wells of plates containing glucose-free RPMI medium without or with supplementation with 20 mM glucose and 10 µg/mL DOX (50 µL/well), for a final concentration of 10 mM glucose and 5 µg/mL DOX, and incubated at 30°C prior to measuring OD_600_ using a CG-12 Cell-Grower Robot, third generation (S&P Robotics) at 24 h.

### Measurement of *C. albicans* ATP content

YPD (5 mL) was inoculated with *C. albicans* GRACE strains and grown overnight at 30°C, shaking. On the next day, cells were subcultured to an OD_600_ of 0.1 in YPD (5 mL) in the absence and presence of 0.05 µg/mL DOX and grown overnight at 30°C, shaking. Overnight cultures were then diluted to an OD_600_ of 0.2 in YPD (10 mL) in the absence and presence of 5 µg/mL DOX and incubated at 30°C, shaking, for 3 h. Cells were pelleted by centrifugation at 2095 × *g* for 1 min, washed three times in PBS, and diluted to an OD_600_ of 0.3 in glucose-free RPMI medium in the absence or presence of 10 µg/mL DOX. Fungal suspension (50 µL/well) was added to the wells of plates containing glucose-free RPMI medium without or with supplementation with 20 mM glucose (50 µL/well), for a final concentration of 10 mM glucose and 5 µg/mL DOX, and incubated for 1 h at 37°C under 5.5% CO_2_. OD_600_ was measured, and 10% trichloroacetic acid (TCA) and 8 mM EDTA were added. Cells were incubated at room temperature for 1 min to enable the extraction of intracellular ATP. Cell debris was pelleted by centrifugation at 3725 × *g* for 2 min, and supernatants were diluted 1:20 into 100 mM Tris-HCl pH 8 with 1 mM EDTA (100 µL/well) in a white, opaque 96-well plate (Nunc, 165306). An equal volume of BacTiter-Glo Reagent (Promega) was added, and luminescence was read immediately using a Tecan Infinite F200 Pro (Tecan). For each well, luminescence was normalized to OD_600_ readings acquired prior to TCA extraction.

### CFSE staining of *C. albicans* cells

J774A.1 cells were diluted to 4 × 10^5^ cells/mL in RPMI medium supplemented with 3% HI-FBS. Cell suspension (100 µL/well) was added to 96-well plates and incubated for 18 h at 37°C under 5.5% CO_2_. For CFSE staining of *C. albicans* cells, all washes and incubations were performed with PBS supplemented with 0.05 µg/mL DOX to maintain target gene repression. *C. albicans* overnight cultures were diluted to an OD_600_ of 0.5, washed twice, and incubated with 5 µg/mL CFSE for 30 min at room temperature in the dark with rotation. CFSE was removed by washing twice with 2% BSA and twice in PBS alone, and cell suspensions were passed through a 26G needle five times to dissociate clumps. Cells were diluted to 2 × 10^5^ cells/mL in RPMI medium supplemented with 3% HI-FBS in the presence of 10 µg/mL DOX and added (100 µL/well) to the wells of plates previously seeded with J774A.1 cells, for a final concentration of 5 µg/mL DOX. Co-cultures were incubated for 4 or 16 h at 37°C under 5.5% CO_2_, fixed and permeabilized, as described above, and incubated with anti-*C*. *albicans* antibody (1:1,000 dilution, Abcam, ab53891) for 1 h at room temperature. The primary antibody was removed, and cells were washed three times in PBS and incubated with anti-rabbit AF555 antibody (1:500 dilution, Thermo Fisher Scientific, A-31572) for 1 h at room temperature. The secondary antibody was removed, cells were washed three times in PBS, and medium was replaced with PBS containing 0.02% sodium azide and 300 ng/mL DAPI. Images were obtained as described above for BMDM infection with *C. albicans*.

### *C. albicans* escape from phagocytes

J774A.1 cells were diluted to 2.5 × 10^5^ cells/mL in RPMI medium supplemented with 3% HI-FBS. Cell suspension (1 mL/well) was added to glass coverslips in 24-well plates (Falcon) and incubated for 18 h at 37°C under 5.5% CO_2_. On the following day, *C. albicans* overnight cultures were diluted to 2 × 10^5^ cells/mL in RPMI medium supplemented with 3% HI-FBS in the absence and presence of 15 µg/mL of DOX. Fungal cell suspension (500 µL/well) was added to the wells of plates previously seeded with J774A.1 cells, for a final concentration of 5 µg/mL DOX. Co-cultures were incubated for 4 or 16 h at 37°C under 5.5% CO_2_ and fixed with 4% methanol-free formaldehyde for 10 min. Formaldehyde was removed, and cells were washed three times with PBS, blocked for 15 min in 2% BSA, and stained with FITC-conjugated anti-*C*. *albicans* antibody (1:200 dilution) for 1 h at room temperature to stain extracellular *C. albicans* cells. The antibody was removed, and cells were washed three times in PBS, permeabilized, blocked, and incubated with anti-*C*. *albicans* antibody (1:200 dilution) for 1 h at room temperature. The primary antibody was removed, and cells were washed three times in PBS and incubated with anti-rabbit AF555 antibody (1:250 dilution) for 1 h at room temperature to stain intracellular and extracellular *C. albicans* cells. The secondary antibody was removed, cells were washed three times in PBS, and medium was replaced with PBS containing 0.02% sodium azide and 300 ng/mL DAPI. Images were obtained using an inverted microscope as described above for BMDM infection with *C. albicans*.

### Phagocyte killing by *C. albicans*

J774A.1 cells were diluted to 4 × 10^5^ cells/mL in RPMI medium supplemented with 3% HI-FBS. Cell suspension (100 µL/well) was added to 96-well plates and incubated for 18 h at 37°C under 5.5% CO_2_. On the following day, *C. albicans* overnight cultures were diluted to 2 × 10^5^ cells/mL in RPMI medium supplemented with 3% HI-FBS and 2 µg/mL PI (Sigma, P4170) in the absence and presence of 10 µg/mL DOX. Fungal cell suspension (100 µL/well) was added to the wells of plates previously seeded with J774A.1 cells, for a final concentration of 1 µg/mL PI and 5 µg/mL DOX, and incubated for 16 h at 37°C under 5.5% CO_2_. Co-cultures were imaged using the IncuCyte S3 Live-Cell Analysis System, and the PI area was quantified using the IncuCyte Basic Analyzer Software.

### Fungal quantitative RT-PCR

Quantitative RT-PCR was carried out as previously described (25) and in Text S1.

### Quantification and statistical analysis

The statistical significance of triplicate measurements was determined using an unpaired two-tailed *t*-test or one-way analysis of variance (Tukey’s multiple comparison test with a *P* ≤ 0.05 considered significant) in GraphPad Prism, version 9. *n* represents the number of times an experiment was run with independent overnight cultures of *C. albicans*. Statistical details for each experiment can be found in the corresponding figure legend.

## Data Availability

The MΦ and TC neural networks are available through the Open Science Foundation (https://osf.io/2q6xf/). Images of the primary screen are available from L.E.C. upon request.

## SUPPLEMENTAL MATERIAL

Text S1 mbio.02745-23-s0001.docx Supplemental figure legends, methods, and references.

Fig. S1 mbio.02745-23-s0002.pdf Classification of *C. albicans* morphology in phagocytes by MΦ-Candescence is sensitive and precise.

Fig. S2 mbio.02745-23-s0003.pdf Classification of *C. albicans* morphology under monoculture in TC conditions by TC-Candescence is sensitive and precise.

Fig. S3 mbio.02745-23-s0004.pdf *C. albicans* relies on respiration for filamentation in phagocytes, but not for viability.

Fig. S4 mbio.02745-23-s0005.pdf Compromise of respiration in the absence of glucose impairs *C. albicans* filamentation and growth under both neutral and acidic pH.

Fig. S5 mbio.02745-23-s0006.pdf RIP1 is important to maintain ATP levels and enable filamentation in the absence of fermentable carbon sources.

Fig. S6 mbio.02745-23-s0007.pdf BAY-876 has no reproducible impact on *C. albicans* growth or filamentation, while 2DDG has no impact on *C. albicans* filamentation but inhibits growth.

Fig. S7 mbio.02745-23-s0008.pdf Treatment of infected macrophages with BAY-876 or 2DDG does not decrease immune cell viability, and SLC16A3 is not essential for intraphagosomal filamentation of *C. albicans*.

Fig. S8 mbio.02745-23-s0009.pdf Biological replicate of the experiment presented in Fig. 5D confirms reproducibility of results.

Supplemental table mbio.02745-23-s0010.xlsx Table S1.

ASM does not own the copyrights to Supplemental Material that may be linked to, or accessed through, an article. The authors have granted ASM a non-exclusive, world-wide license to publish the Supplemental Material files. Please contact the corresponding author directly for reuse.
