# Supplementary material for: Respiration supports intraphagosomal filamentation and escape of Candida albicans from macrophages
Source: mBio. 2023 Dec 1;14(6):e02745-23. doi: 10.1128/mbio.02745-23 (PMC10746240; doi:10.1128/mbio.02745-23)
Supplement: Text S1 — Supplemental figure legends, methods, and references. [file mbio.02745-23-s0001.docx]

**Figure Legends**

**Figure S1. Classification of *C. albicans* morphology in phagocytes by MΦ-Candescence is sensitive and precise. A)** Example annotations of objects detected by MΦ-Candescence in images of *C. albicans* in co-culture with mouse MΦ cells. Uninfected MΦ (brown) denotes all uninfected phagocytes. Classes 0-3 represent fungal cells that have been internalized by phagocytes (infected). Classes 0 and 1 together represent all non-filamentous cells, whereas classes 2 and 3 represent filamentous cells. Also included are examples of an object in the field of view that was not able to be classified (dark blue), an artifact (purple), and a scale bar (green). **B)** Image on the left is a typical field of view which has been manually annotated with the location and class of each object. The image on the right illustrates predictions by MΦ-Candescence for that same image, which is from the validation dataset. **C)** Confusion matrix for MΦ-Candescence across the validation dataset. Here the accuracy is computed as a function of the number of correct non-filamentous and filamentous predictions and the number of mistakes, which includes either incorrect classifications of filamentous cells as non-filamentous (red), non-filamentous cells as filamentous (pink), or false negative predictions corresponding to cells of classes 0-3 that were classified as either uninfected MΦ, unknown, or artifacts (orange) and uninfected MΦ, unknown, or artifacts that were assigned a class from 0-3 (purple). Sensitivity and precision measure the rate of false negatives and false positives, respectively, and F1 combines sensitivity and precision into a single summary statistic. **D)** Scatter plot depicting the correlation between the average fraction of non-filamentous cells for each GRACE strain in phagocytes in replicate 1 versus replicate 2.

**Figure S2. Classification of *C. albicans* morphology under monoculture in TC conditions by TC-Candescence is sensitive and precise. A)** Example annotations of objects detected by TC-Candescence in images of *C. albicans* in monoculture. Classes 0 and 1 together represent non-filamentous cells whereas classes 2 and 3 represent filamentous cells. Also included are examples of an object in the field of view that was not able to be classified (dark blue), an artifact (purple), and a scale bar (green). **B)** Image on the left is a typical field of view which has been manually annotated with the location and class of each object. The image on the right illustrates predictions by TC-Candescence for that same image, which is from the validation dataset. **C)** Confusion matrix for TC-Candescence across the validation dataset. Accuracy is computed as a function of the number of correct non-filamentous and filamentous predictions and the number of mistakes, which includes either incorrect classifications of filamentous cells as non-filamentous (red), non-filamentous cells as filamentous (pink), or false negative predictions corresponding to cells of classes 0-3 that were classified as either unknown or artifacts (orange) and unknown or artifacts that were assigned a class from 0-3 (purple). Sensitivity, precision, and F1 are as described in Figure S1.  **D)** Scatter plot depicting the correlation between the average fraction of non-filamentous cells for each GRACE strain in monoculture under TC conditions in replicate 1 versus replicate 2.

**Figure S3. *C. albicans* relies on respiration for filamentation in phagocytes, but not for viability. A)** High DOX (5 µg/mL) significantly represses target gene expression in GRACE mutants while having no impact on the expression of the same genes in the wild-type control strain. GRACE strains were grown overnight in the presence or absence of low DOX (0.05 µg/mL) then sub-cultured in the absence or presence of high DOX for 4 hours prior to flash freezing for RNA extraction and measurement of transcript abundance by qRT-PCR. Error bars indicate standard error for technical triplicates. Statistical significance was calculated using a two-sided unpaired *t*-test. ^∗∗^*P* ≤ 0.01. Significance is reported for strains demonstrating a significant decrease in expression in the presence of DOX in two biological replicates. **B)** Repression of genes encoding subunits of the mitochondrial ribosome (*MRP21*), electron transport chain (*COR1*), or AMP-activated protein kinase (*SNF1*) are not required for viability after 4-hour co-culture with phagocytes. GRACE strains were grown overnight in the absence or presence of low DOX prior to co-culture with phagocytes for 4 hours in the absence or presence of high DOX. The inocula used to initiate co-cultures were serially diluted 5-fold in PBS and spotted onto YPD agar. At 4 hours, phagocytes were lysed and internalized *C. albicans* cells, which were released, were harvested and similarly serially diluted and spotted onto YPD agar. Controls are wild type (GRACE library parent), a GRACE strain for an essential gene (*CCT7*), and a GRACE strain for a non-essential gene (*BCR1*). **C)** Repression of *MRP21*, *COR1*, or *SNF1*, results in significant reduction in the intraphagosomal filament length of *C. albicans* within RAW264.7 cells. GRACE strains were grown overnight in the presence of low DOX prior to co-culture with phagocytes in the presence of high DOX. Intraphagosomal filament length was measured 3 hours post-infection. Data are presented as violin plots, where the middle solid line indicates the median and lower and upper dashed lines indicate the first and third quartiles, respectively. Data were analyzed using one-way ANOVA (Tukey's multiple comparisons test) to compare means of four groups. ^∗∗∗∗^*P* ≤ 0.0001. Data are representative of two biological replicates (n=2).

**Figure S4. Compromise of respiration in the absence of glucose impairs *C. albicans* filamentation and growth under both neutral and acidic pH. A-B)** *MRP21*, *COR1*, and *SNF1* are needed for filamentation in the absence of glucose under neutral pH. GRACE strains and the corresponding wild-type control were grown overnight in the presence or absence of low DOX prior to culture for 4 hours in glucose-free RPMI medium without or with supplementation with glucose (10 mM) and in the absence or presence of high DOX. Filament length was measured using the IncuCyte NeuroTrack Processing Module. Error bars indicate standard deviation for technical triplicates, and statistical significance was calculated using a two-sided unpaired *t*-test. ^∗∗^*P* ≤ 0.01; ^∗∗∗^*P* ≤ 0.001. Significance is reported for strains demonstrating a significant decrease in filament length in the presence of DOX. Data are a biological replicate of the experiment presented in Figure 3C and 3D. **C-D)** *MRP21*, *COR1*, and *SNF1* are needed for filamentation in the absence of glucose under acidic pH (pH 5.5). GRACE strains and the corresponding wild-type control were grown overnight in the presence or absence of low DOX prior to culture for 4 hours in glucose-free RPMI medium without or with supplementation with glucose (10 mM) and in the absence or presence of high DOX. **E-F)** *MRP21*, *COR1*, and *SNF1* are needed for growth in the absence of glucose under neutral **(E)** and acidic **(F)** pH. GRACE strains and the corresponding wild-type control were grown overnight in the presence or absence of low DOX prior to culture for 24 hours at 30 °C in glucose-free RPMI medium in the absence or presence of high DOX. Error bars indicate standard deviation for technical triplicates, and statistical significance was calculated using a two-sided unpaired *t*-test. ^∗^*P* ≤ 0.05; ^∗∗^*P* ≤ 0.01; ^∗∗∗^*P* ≤ 0.001. Significance is reported for strains demonstrating a significant decrease in growth in the presence of DOX.

**Figure S5. *RIP1* is important to maintain ATP levels and enable filamentation in the absence of fermentable carbon sources. A)** The requirement for *RIP1*, a nuclearly-encoded subunit of electron transport chain complex III, to maintain fungal ATP levels is increased in the absence of glucose. Strains were grown overnight, sub-cultured for 3 hours, then cultured in glucose-free RPMI medium without or with supplementation with glucose (10 mM). After an hour, ATP level was measured by luciferase-based luminescence assay and normalized by relative cell number in the well (OD_600_). **B-C)** *RIP1* is required for filamentation in the absence of glucose. Strains were grown overnight, then cultured for 4 hours in glucose-free RPMI medium without or with supplementation with glucose (10 mM). **B)** Images of wild-type and mutant strains. **C)** Quantification of filament length (mm/mm^2^) in cultures of the strains from the images shown in **B** measured using the IncuCyte NeuroTrack Processing Module. For **A** and **C**, error bars indicate SD for technical triplicates. Statistical significance was calculated using a two-sided unpaired *t*-test. ^∗^*P* ≤ 0.05; ^∗∗^*P* ≤ 0.01; ^∗∗∗^*P* ≤ 0.001, ^∗∗∗∗^*P* ≤ 0.0001. Significance is reported for decreases in filament length or ATP level in both biological replicates. Data are representative of two biological replicates (n=2). **D-G)** Biological replicate of data presented in Figure 3E and 3F confirms reproducible results. GRACE strains were grown overnight in the presence or absence of low DOX, sub-cultured, grown for 3 hours with or without high DOX, then cultured in glucose-free RPMI medium without (**D-E**) or with supplementation with glucose (10 mM) (**F-G**) in the absence or presence of high DOX. After an hour, ATP levels were measured by luciferase-based assay and normalized by relative cell number in the well (OD_600_). Error bars indicate standard deviation of technical triplicates, and statistical significance was calculated using a two-sided unpaired *t*-test. ^∗^*P* ≤ 0.05; ^∗∗^*P* ≤ 0.01; ^∗∗∗^*P* ≤ 0.001. Significance is reported for strains demonstrating a significant decrease in ATP level in the presence of DOX.

**Figure S6. BAY-876 has no reproducible impact on *C. albicans* growth or filamentation, while 2DDG has no impact on *C. albicans* filamentation but inhibits growth. A)** BAY-876 (5 µM) has no impact on *C. albicans* filamentation after 4-hour culture at 37 °C under 5% CO_2_ in glucose-free RPMI adjusted to neutral or acidic pH (pH 5.5). **B)** BAY-876 has no reproducible impact on *C. albicans* growth after 24-hour culture at 30°C in glucose-free RPMI adjusted to neutral or acid pH. Error bars indicate standard deviation for technical triplicates, and statistical significance was calculated using a two-sided unpaired *t*-test. ^∗^*P* ≤ 0.05. **C)** 2DDG (10 mM) has no impact on *C. albicans* filamentation after 4-hour culture at 37°C under 5% CO_2_ in glucose-free RPMI supplemented with glucose (10 mM), adjusted to neutral or acidic pH. **D)** 2DDG results in a significant decrease in *C. albicans* growth after 24-hour culture at 30°C in glucose-free RPMI supplemented with glucose (10 mM) adjusted to neutral or acid pH. Error bars indicate standard deviation for technical triplicates, and statistical significance was calculated using a two-sided unpaired *t*-test. ^∗∗^*P* ≤ 0.01; ^∗∗∗^*P* ≤ 0.001.

**Figure S7. Treatment of infected macrophages with BAY-876 or 2DDG does not decrease immune cell viability, and SLC16A3 is not essential for intraphagosomal filamentation of *C. albicans*. A)** RAW264.7 cells cultured in the absence (uninfected) or presence (infected) of *C. albicans*. After 45 minutes, cultures were washed and medium replaced with: RPMI, RPMI with BAY-876 (5 µM), or RPMI with 2DDG (10 mM), as indicated. Phagocyte viability was assessed after a further 3-hour incubation. **B)** RAW264.7 cells were incubated with *C. albicans* in RPMI for 45 minutes to allow phagocytosis to occur then co-cultures were treated with vehicle (DMSO), Bindarit (100 µM), or BAY-8002 (100 µM). Intraphagosomal filament length of *C. albicans* was measured at 3 hours post-infection. **C)** *C. albicans* was grown under monoculture conditions in RPMI medium supplemented with vehicle (DMSO), Bindarit (100 µM), or BAY-8002 (100 µM), and filament length was measured after 4 hours. **D)** qPCR was used to examine SLC16A3 transcript levels in RAW264.7 cells. The relative expression of SLC16A3 was determined by normalizing the Ct values to the reference gene *Abt1*, and the data is presented as the percentage of SLC16A3 expression compared to the control group (Ctrl siRNA). **E)** Intraphagosomal filament length of *C. albicans* in SLC16A3-silenced/knockout RAW264.7 cells was measured 4 hours post-infection in standard RPMI. For **A-E**, data were analyzed using a one-way ANOVA (Tukey's multiple comparisons test) to compare means. ns = *P* > 0.05; ^∗^*P* ≤ 0.05; ^∗∗^*P* ≤ 0.01; ^∗∗∗^*P* ≤ 0.001, ^∗∗∗∗^*P* ≤ 0.0001. For **B**, **C**, and **E**, data are presented as violin plots, where the middle solid line indicates the median and lower and upper dashed lines indicate the first and third quartiles, respectively. For **A-C** and **E**, data are representative of three biological replicates (n=3).

**Figure S8. Biological replicate of experiment presented in Figure 5D confirms reproducibility of results. A-B)** Quantification of phagocyte killing by wild-type and mutant *C. albicans* strains in the absence or presence of DOX. Error bars indicate standard deviation for technical triplicates, and statistical significance was calculated using a two-sided unpaired *t*-test. ^∗^*P* ≤ 0.05; ^∗∗^*P* ≤ 0.01.

**Table S1. Manual scoring and Candescence parameters for GRACE strains analyzed for their filamentation phenotype in phagocytes and under TC conditions. A)** All GRACE strains and their associated parameters. **B)** Hits identified by manual and computational annotation of GRACE strain filamentation phenotype in phagocytes and their associated parameters. **C)** GO- enrichment of the genes required for filamentation in phagocytes identified manually and/or by Candescence. **D)** Hits identified by comparing manual and computational annotation of GRACE strain filamentation phenotype in phagocytes to their filamentation phenotype in monoculture under TC conditions, and their associated parameters. **E)** GO-enrichment of the genes required for filamentation in phagocytes but not in monoculture under TC conditions identified manually and/or by Candescence. **F)** The total number of predicted objects by Candescence algorithms across all MΦ and TC functional genomic screen images. **G)** Strains used in this study. **H)** Primers used in this study.

**Text S1**

**Strain construction**

**CaLC5837.** This strain with both copies of C3_04430W_A or *RIP1* deleted was made using a transient CRISPR approach adapted from Min *et al*. 2016 (5). sgRNA was generated using gene-specific primers oLC7458 and oLC7459 and universal primers oLC6926, oLC6927, oLC6928, and oLC6929 (Table S1H) from plasmid pLC1081 (pV1093 from Min *et al*. 2016) (5). The *CaCAS9* cassette was amplified from pLC1081 using primers oLC6924 and oLC6925 (Table S1H). Repair template was amplified from plasmid pLC1101 (pFA-HA-HIS1 from Lavoie *et al*. 2008) (6) using primers oLC7455 and oLC6933 (Table S1H). The repair template, sgRNA, and Cas9 DNA were transformed into CaLC239 (SN95). Lack of a wild-type allele was confirmed using genotyping PCR using primers oLC7456 and oLC7457 (Table S1H).

**Preparation of bone marrow-derived macrophages.** For Figure 3A, Bone marrow-derived macrophages (BMDMs) were generated by culturing bone marrow cells isolated from the femur and tibia of 6- to 8-week-old healthy female C57BL/6J mice (The Jackson Laboratory, JAX_000664). For differentiation, 5 × 10^6^ cells were seeded into a 10 cm^2^ Petri dish (VWR) in RPMI medium supplemented with 10% HI-FBS, 25 ng/mL M-CSF, 100 U/L penicillin-streptomycin, and 2 mM L-glutamine and incubated for seven days at 37 °C and 5.5% CO_2_. Adherent cells were detached by incubating dishes in 10 mL ice-cold PBS for 10 min at 4°C then gently pipetting the PBS across the dish. For Figure 4C, the bone marrow of C57BL/6J mice was extruded and centrifuged at 15,000 × g for 10 sec into cold PBS, washed once in sterile distilled water to lyse red blood cells, and pelleted at 500 × g for 10 sec into cold PBS. Pellets were suspended in DMEM with 10% HI-FBS, 10 ng/mL M-CSF, 1x antibiotic-antimycotic solution, and plated at a density of 4 x 10^5^ cells per 10 cm^2^ Petri dish for 5-8 days before use.

**Knockout of monocarboxylate transporter SLC16A3 in RAW264.7 cells.** A custom ID plasmid from Sigma (vector p01, gene ID 80879, sequence TGAGTGTCTTCCGAGACCG) was transfected into RAW264.7 cells using FuGENE HD (Promega) according to manufacturer’s instruction. Transfected cells were sorted for GFP-positive and propidium iodide-negative cells using fluorescence-activated cell sorting, and subsequently expanded in RPMI supplemented with 10% HI-FBS.

**Silencing of** **monocarboxylate transporter SLC16A3 in RAW264.7 cells.** SLC16A3 (mouse, Dharmacon, L061813-0005) or control siRNA from (Dharmacon, D-001810-10-20) were used for silencing in RAW264.7 macrophages. Cells from 1 well of a confluent 6-well plate were scraped, centrifuged, and resuspended in R buffer (220 μL). siRNA (10 μL) was added to R buffer (110 μL) with RAW264.7 cells and electroporated with 2 pulses (20 ms) at 1300. The NEON Transfection System (Invitrogen, MPK5000) and NEON Transfection Kit (Invitrogen, MPK10096K) were used for electroporation of siRNA into the RAW264.7 macrophages. Approximately 200,000 electroporated RAW264.7 cells were added to 1 well of a 12-well plate with 18 mm coverslip containing DMEM supplemented with 10% HI-FBS. Electroporated RAW264.7 cells were incubated overnight at 37^o^C with 5.5% CO_2_. Silenced RAW264.7 cells were validated by qPCR with a custom-made TaqMan Gene Expression Assay (Slc16a3 FAM, Thermo Fisher Scientific).

***C. albicans* viability.** J774A.1 cells were diluted to 2.5 x 10^5^ cells/mL in RPMI medium supplemented with 3% HI-FBS. Cell suspension (100 µL/well) was added to 96-well plates and incubated for 18 h at 37°C under 5.5% CO_2_. The following day, *C. albicans* overnight cultures were diluted to an OD_600_ of 0.05 in RPMI medium supplemented with 3% HI-FBS in the absence or presence of 15 µg/mL DOX. Fungal cell suspension (50 µL/well) was added to the wells of plates previously seeded with J774A.1 cells, for a final concentration of 5 µg/mL DOX, and incubated for 4 h at 37°C under 5.5% CO_2_. To determine *C. albicans* viability prior to co-culture with phagocytes, fungal cell suspensions were serially diluted 5-fold in PBS and spotted onto YPD agar plates. To determine *C. albicans* viability after co-culture with phagocytes, supernatant was removed, co-cultures were incubated with 0.1% Triton X-100 for 15 min at 4°C to lyse phagocytes, then lysates were serially diluted 5-fold in PBS and spotted onto YPD agar plates. Plates were imaged after incubation for 36 h at 30°C.

**Fungal qRT-PCR.** *C. albicans* overnight cultures were subcultured to an OD_600_ of 0.1 in YPD medium (10 mL) in the presence and absence of 5 µg/mL DOX and grown for 4 h to mid-log phase before cell pellets were collected, flash frozen, and stored at -80°C. Cells were lysed by bead beating and RNA was extracted using the RNAeasy Kit (Qiagen). Samples were DNAse treated using the DNA-free DNA Removal Kit (Invitrogen), and cDNA was amplified using the iScript cDNA Synthesis Kit (Bio-Rad). PCR was performed using the Fast SYBR Green Master Mix (Applied Biosystems) and the Bio-Rad CFX-384 Real-Time System with the following cycling conditions: 95°C for 3 min, 95°C for 10 sec, and 60°C for 30 sec, for 40 cycles. Reactions were performed in technical triplicate for two biological replicates and data were analyzed using the Bio-Rad CFX Manager 3.1. qRT-PCR primers are listed in Table S1H.

**Candescence**

**Classifiers of *Candida albicans* morphology.** We developed two classifiers using our Candescence approach (7) from images of *C. albicans* in co-culture with a mouse macrophage-like cell line (MΦ) and images of *C. albicans* grown in standard tissue culture (TC) conditions. Candescence was designed using a two-stage object-detection/object classification approach; the software first locates all putative cells (*C. albicans* or phagocyte) in the image and then classifies each such object according to a predefined set of morphologies. Candescence exploits a full convolutional one-stage (FCOS) object detector (8), a deep learning architecture with fast and accurate performance. In the previous effort, Candescence was trained to detect and classify individual *C. albicans* cells into one of nine different morphologies using microscopy images. Both classifiers developed here exploited transfer learning where training was started with the neural network underlying the original Candescence.

**The phagocyte classifier: MΦ-Candescence.** The first classifier was built across 6,714 successful images covering the 3,332 GRACE strains and controls grown in co-culture with mouse MΦ cells. 82 images were randomly chosen (1.2% of all images) for manual annotation using Labelbox annotation software (https://labelbox.com). We labelled phagocytes that had not internalized *C. albicans* cells as ‘uninfected’, while phagocytes with internalized *C. albicans* cells were labelled class 0, 1, 2, or 3 based on the degree of filamentation of the internalized fungal cells. *C. albicans-*containing phagocytes were labelled class 0 if there was no evidence of filamentation (the fluorescence was only a dot), and labelled class 1 if there was evidence of a filamentous structure. Class 2 was reserved for macrophages with extensive fluorescence and filamentation with lengths comparable to wild type. Class 3 was used to denote macrophages where there was evidence of multiple *C. albicans* cells with almost complete fluorescence across its area. Other non-phagocyte or *C. albicans* objects (slide scratches, smears, and dust) were labelled as artifacts. *C. albicans* cells were labelled as unknown if it was not possible to determine their class, for example, in cases where a portion of the object lay outside the field of view. Scales bars that were included in each image by the imaging platform where also labelled. Although artifacts, scale bars, and unknowns were included in the training, they were not included in the downstream analyses (Figure S1A).

57 of the 82 images were assigned to the training dataset with the remaining 25 assigned to validation dataset. A further test dataset was designed using an additional 10 images manually labelled by the authors. Only the training and validation datasets were used during the training phase of MΦ-Candescence; the test set was used as an independent measure of performance after training, following the strategy outlined in the original paper.

A hyperparameter search was carried out over several variables. We found that transfer learning from Candescence version 1.0 improved performance. Complete freezing, meaning that no layer of the original Candescence version 1.0 neural network was allowed to change optimized performance; here only additional new layers that process the input image were allowed to vary. Best performance across standard learning parameters included 0.01 for the learning rate, 0.9 for momentum, 0.001 for decay and the number of epochs, which is the number of complete cycles through the learning and validation datasets, was just below 1000. Figure S1B provides an example of a manually annotated image from the training set and the annotations predicted by MΦ-Candescence. Figure S1C depicts the confusion matrix across the validation dataset with the performance across classification and object detection, including false positive object predictions termed hallucinations (since they represent objects that MΦ-Candescence predicts to exist, but which are not present in the ground truth images) and false negative blind spots (since MΦ-Candescence did not locate the objects). The hyperparameter search for the classifier also requires the determination of a suitable threshold 𝛕, which specifies a threshold on the probability obtained from the softmax output layer of the FCOS that a class prediction must exceed, if MΦ-Candescence is to accept that class label for an object. Our analysis suggested performance was maximized at 𝛕=0.2. Figure S1D depicts the correlation between the average fraction of non-filamentous cells for each GRACE strain in phagocytes in replicate 1 versus replicate 2.

**The tissue-culture classifier: TC-Candescence**. The second classifier was trained using images of *C. albicans* grown in monoculture under standard TC conditions. The goal of the classifier is to correctly identify and label each detected *C. albicans* based on their degree of filamentation. 98 images were randomly chosen (1.5% of all images) from a total of 6,714 images covering the 3,332 GRACE strains and controls. A few strains and controls lacked images of sufficient quality for the learner and approximately 100 strains did not have a second replicate. Proceeding as above, our team manually located every *C. albicans* cell in the images and assigned it a label from class 0 to class 3. Class 0 corresponds to *C. albicans* cells in the yeast state, class 1 corresponds to cells that exhibit the first stages of filamentation, class 2 corresponds to cells exhibiting wild-type levels of filamentation, and class 3 corresponds to irresolvable clusters of overlapping filamentous cells. As above, unknown and artifact classes were used to annotate technical errors and cases where we were uncertain of the class, respectively (Figure S2A). One individual (MH) examined all 98 images to ensure as best possible a uniform and consistent markup of the images across labellers.

71 of the 98 images were assigned to the training dataset with the remaining 27 assigned to validation dataset. A further test dataset was designed using an additional 10 images manually labelled by the authors. Only the training and validation datasets were used to train TC-Candescence; the test set was used as an independent measure of performance after training, following the strategy outlined in the original paper.

A hyperparameter search was carried out over the same several variables as above. Again, transfer learning was deemed to improve performance. One layer of freezing induced the best performance. Standard search parameters included the learning rate (best performance 0.01), momentum (0.99), and decay (0.001). The number of epochs was determined to be 1000 after using standard techniques to avoid over-learning. This compares only objects that were both identified in the manually annotated images and predicted by TC-Candescence. Figure S2B provides an example of a manually annotated image from the training set and the annotations predicted by TC-Candescence. Figure S2C further breaks down performance including hallucinations and blind spots. The classification threshold 𝛕 yielded best results at 𝛕=0.3. Figure S2D depicts the correlation between the average fraction of non-filamentous cells for each GRACE strain in monoculture under TC conditions in replicate 1 versus replicate 2.

Both the MΦ and TC hyperparameter searches required approximately one month of constant computation with 10 GeForce NVIDIA 1080 Ti GPUs. Table S1A provides the list of all strains and their associated parameters.

**Supplemental References**

1. Roemer T, Jiang B, Davison J, Ketela T, Veillette K, Breton A, Tandia F, Linteau A, Sillaots S, Marta C, Martel N, Veronneau S, Lemieux S, Kauffman S, Becker J, Storms R, Boone C, Bussey H. 2003. Large-scale essential gene identification in *Candida albicans* and applications to antifungal drug discovery. Mol Microbiol 50:167–181.

2. Fu C, Zhang X, Veri AO, Iyer KR, Lash E, Xue A, Yan H, Revie NM, Wong C, Lin ZY, Polvi EJ, Liston SD, VanderSluis B, Hou J, Yashiroda Y, Gingras AC, Boone C, O’Meara TR, O’Meara MJ, Noble S, Robbins N, Myers CL, Cowen LE. 2021. Leveraging machine learning essentiality predictions and chemogenomic interactions to identify antifungal targets. Nat Commun 12:6497.

3. Noble SM, Johnson AD. 2005. Strains and strategies for large-scale gene deletion studies of the diploid human fungal pathogen *Candida albicans*. Eukaryot Cell 4:298–309.

4. Westman J, Walpole GFW, Kasper L, Xue BY, Elshafee O, Hube B, Grinstein S, Walpole G, Kasper L, Xue B, Elshafee O, Hube B, Grinstein S. 2020. Lysosome fusion maintains phagosome integrity during fungal infection. Cell Host Microbe 28:798-812.e6.

5. Min K, Ichikawa Y, Woolford CA, Mitchell AP. 2016. *Candida albicans* gene deletion with a transient CRISPR-Cas9 system. mSphere 1:e00130-16.

6. Lavoie H, Sellam A, Askew C, Nantel A, Whiteway M. 2008. A toolbox for epitope-tagging and genome-wide location analysis in *Candida albicans*. BMC Genomics 9:578.

7. Bettauer V, Costa ACBP, Omran RP, Massahi S, Kirbizakis E, Simpson S, Dumeaux V, Law C, Whiteway M, Hallett MT. 2022. A deep learning approach to capture the essence of *Candida albicans* morphologies. Microbiol Spectr 10:e0147222.

8. Tian Z, Shen C, Chen H, He T. 2019. FCOS: fully convolutional one-stage object detection. Proc IEEE Int Conf Comput Vis 2019-Oct:9626–9635.
