# Supplementary figures and images for: Respiration supports intraphagosomal filamentation and escape of Candida albicans from macrophages
Source: mBio. 2023 Dec 1;14(6):e02745-23. doi: 10.1128/mbio.02745-23 (PMC10746240; doi:10.1128/mbio.02745-23)

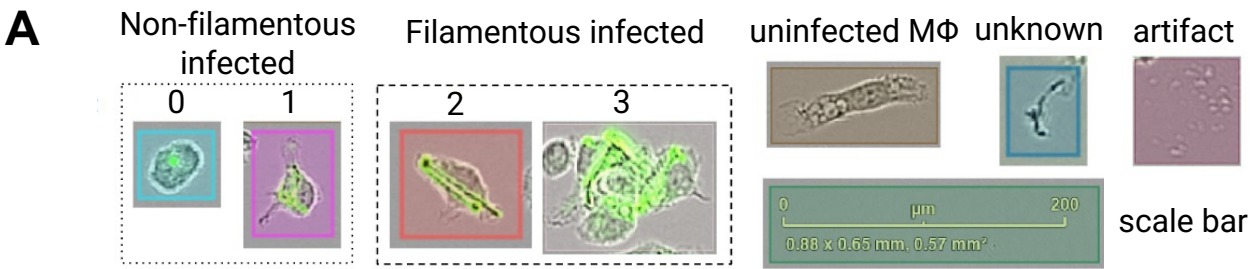

Supplement: Fig. S1 — Classification of C. albicans morphology in phagocytes by MΦ-Candescence is sensitive and precise. [file mbio.02745-23-s0002.pdf]

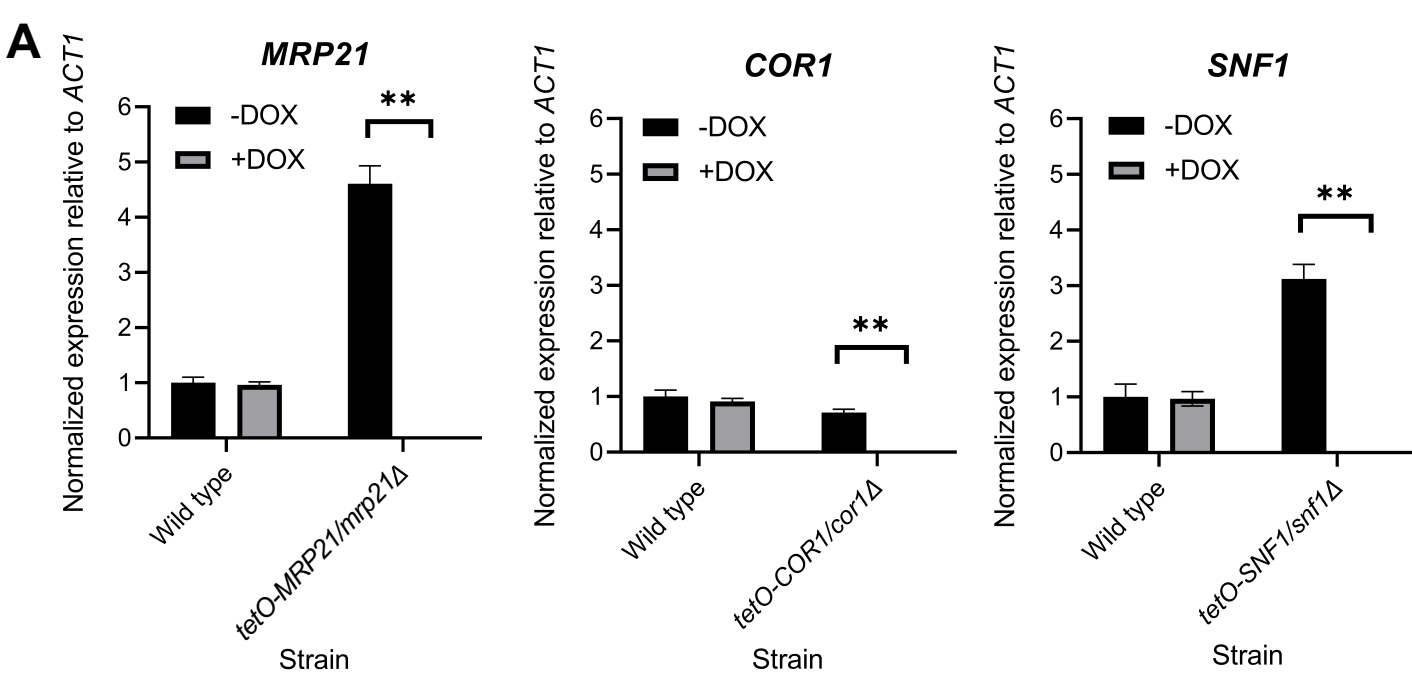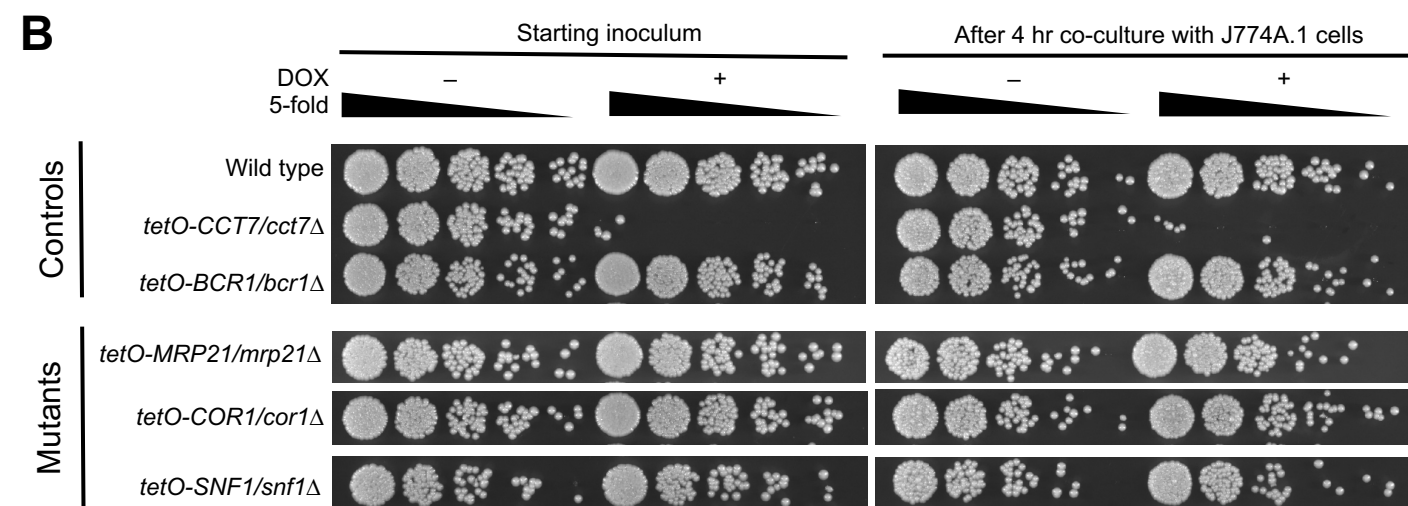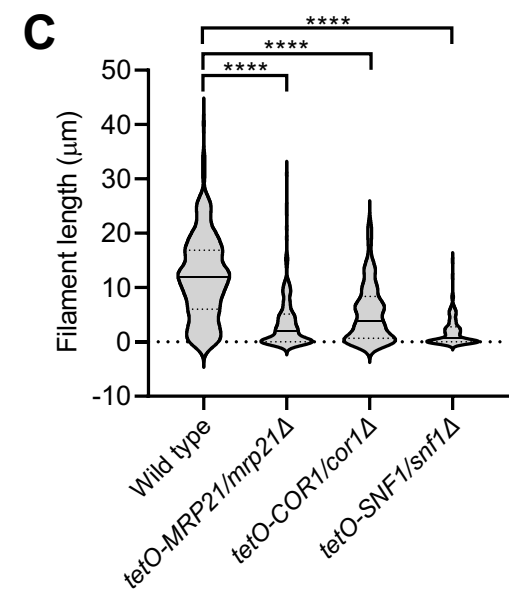

Supplement: Fig. S3 — C. albicans relies on respiration for filamentation in phagocytes, but not for viability. [file mbio.02745-23-s0004.pdf]

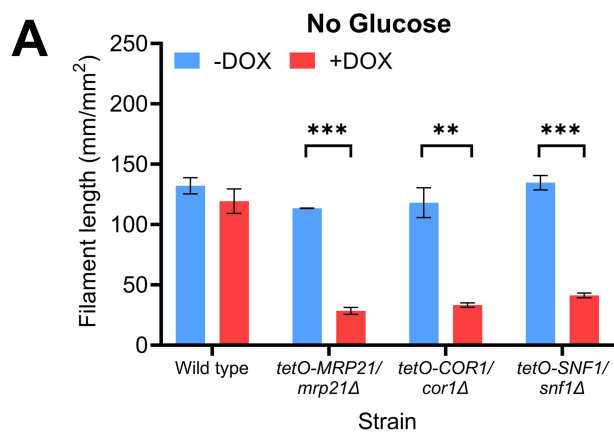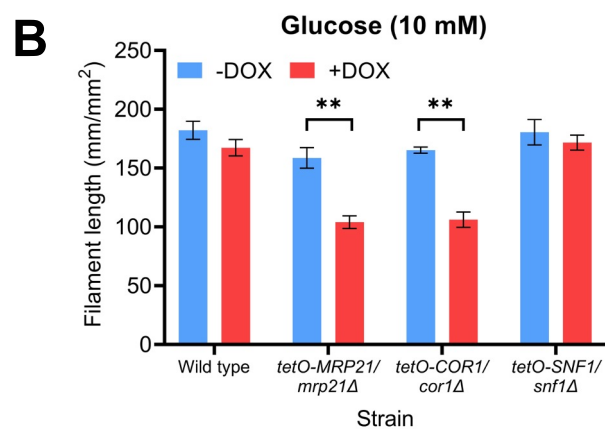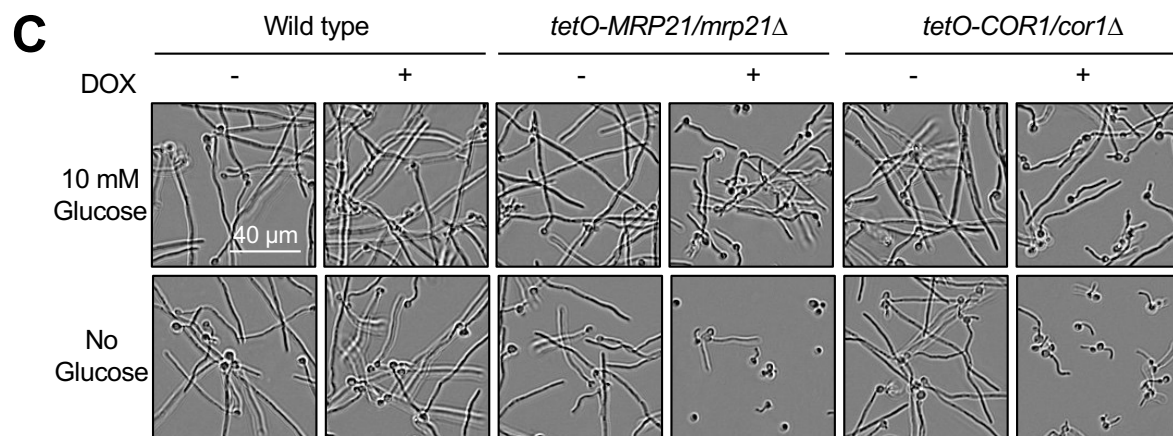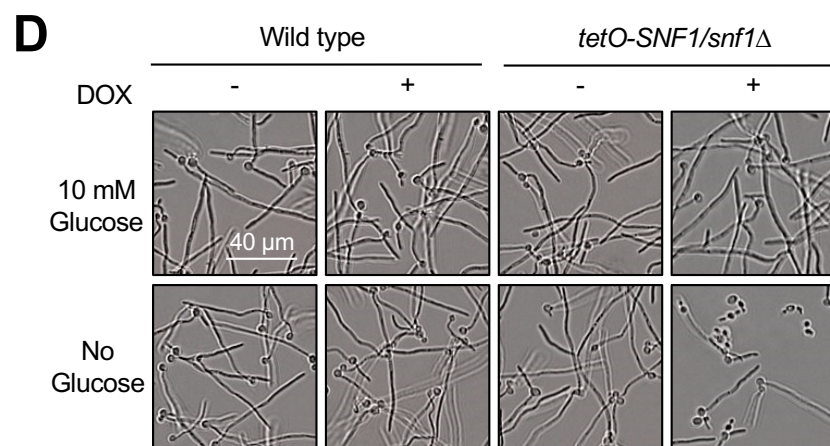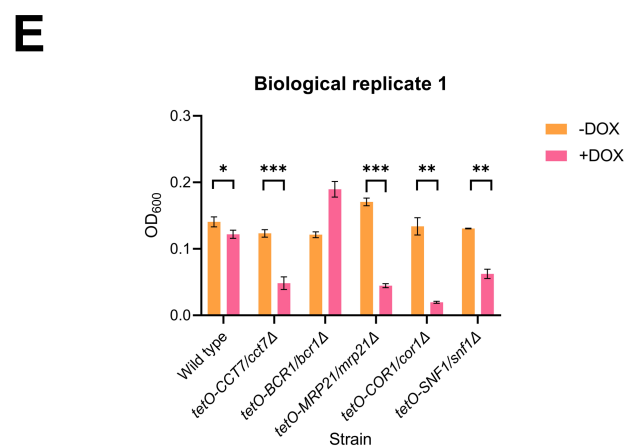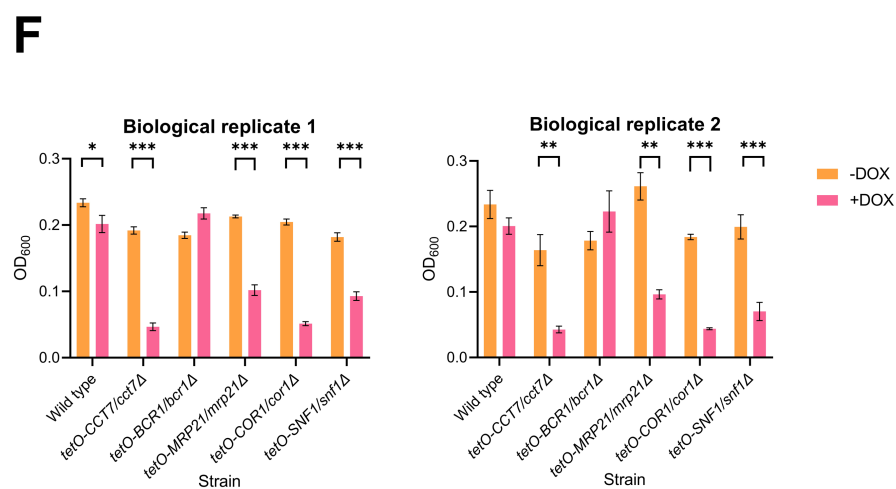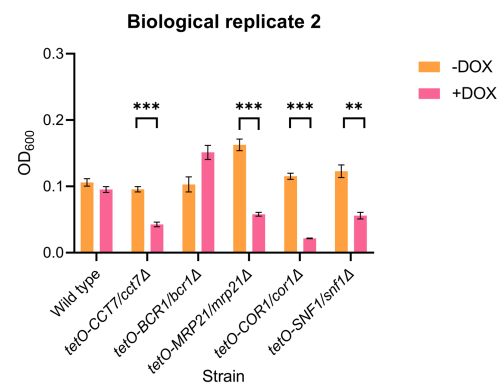

Supplement: Fig. S4 — Compromise of respiration in the absence of glucose impairs C. albicans filamentation and growth under both neutral and acidic pH. [file mbio.02745-23-s0005.pdf]

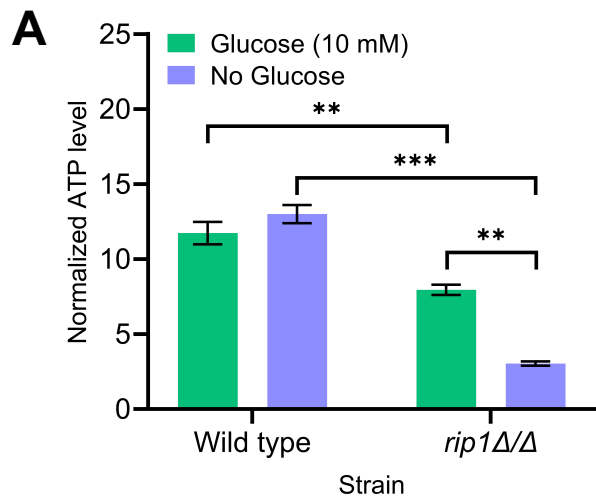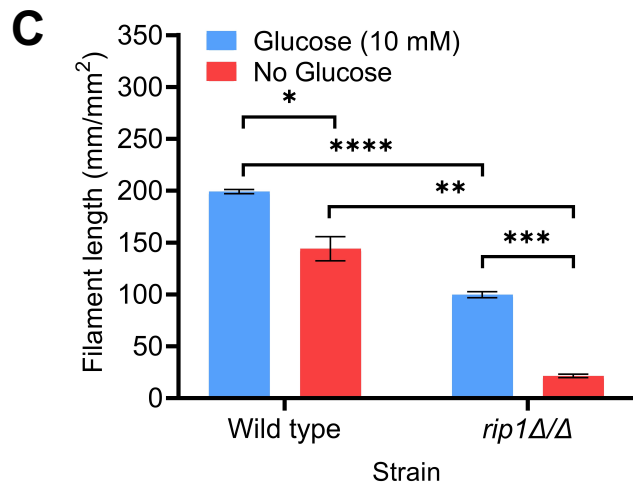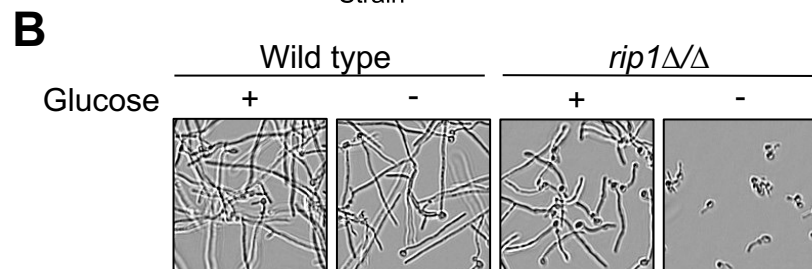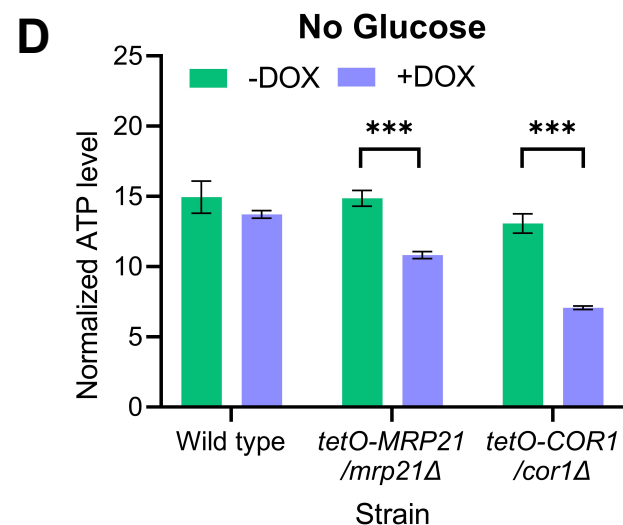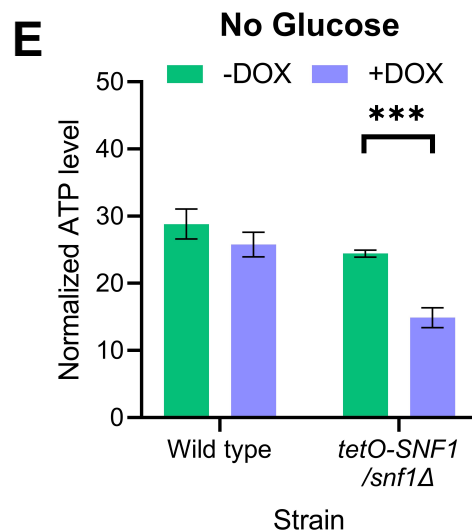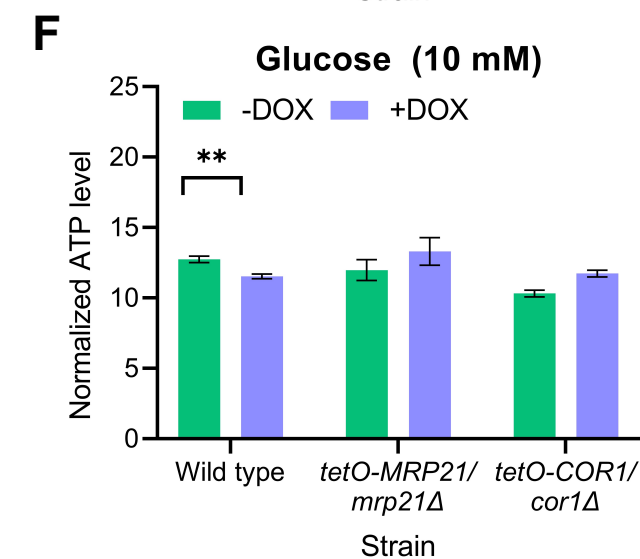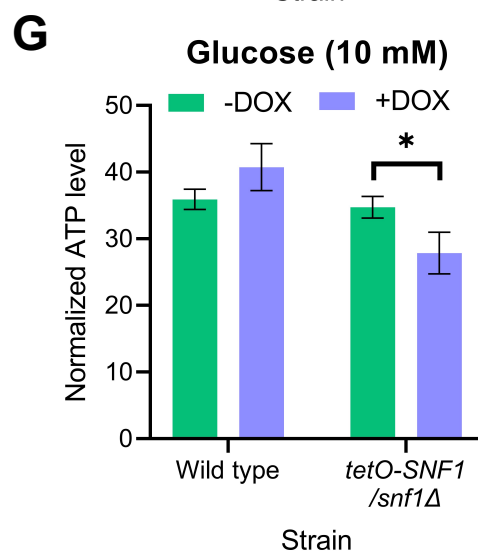

Supplement: Fig. S5 — RIP1 is important to maintain ATP levels and enable filamentation in the absence of fermentable carbon sources. [file mbio.02745-23-s0006.pdf]

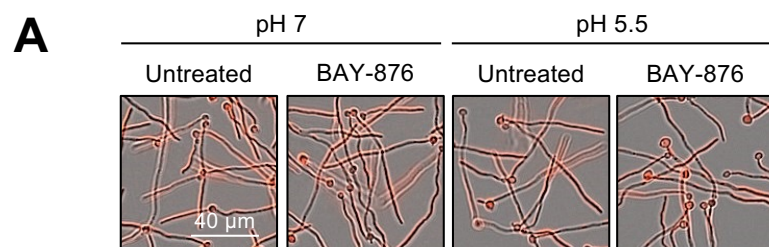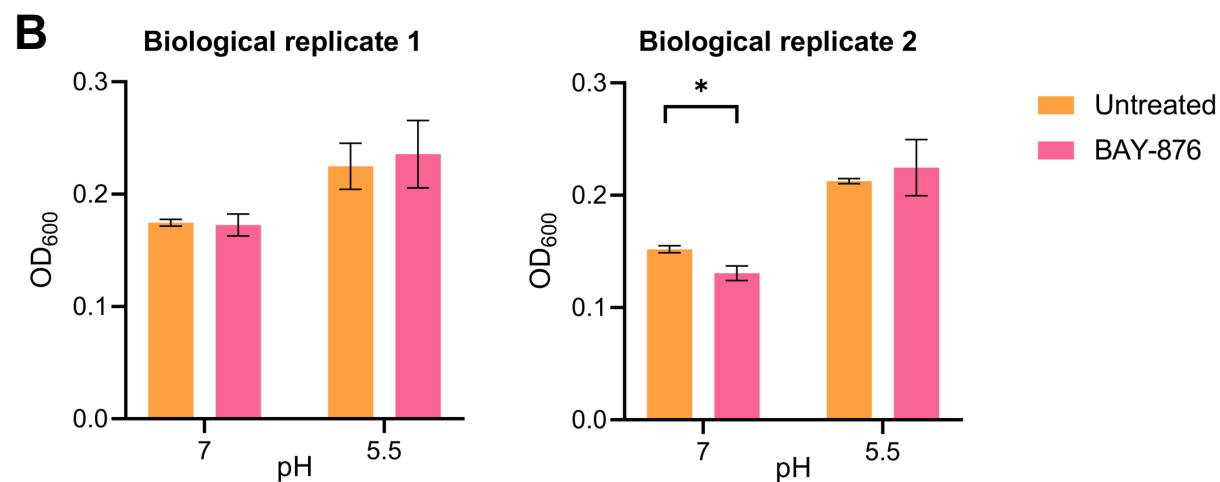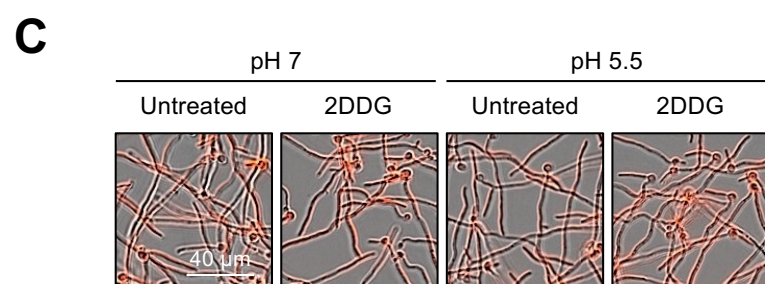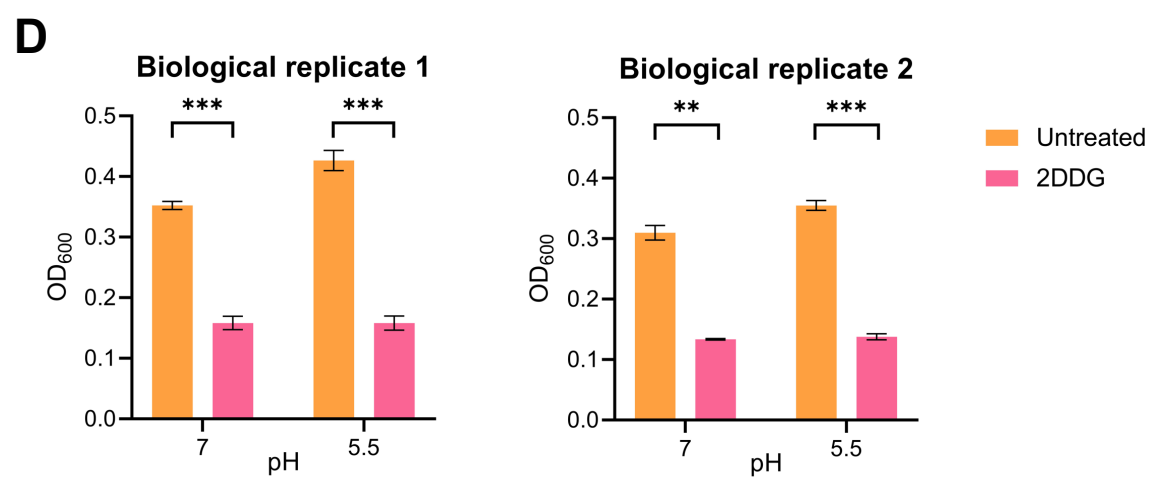

Supplement: Fig. S6 — BAY-876 has no reproducible impact on C. albicans growth or filamentation, while 2DDG has no impact on C. albicans filamentation but inhibits growth. [file mbio.02745-23-s0007.pdf]

**A**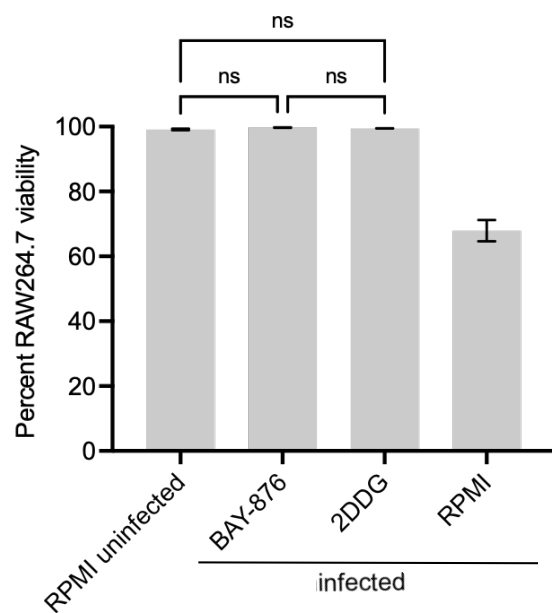**B**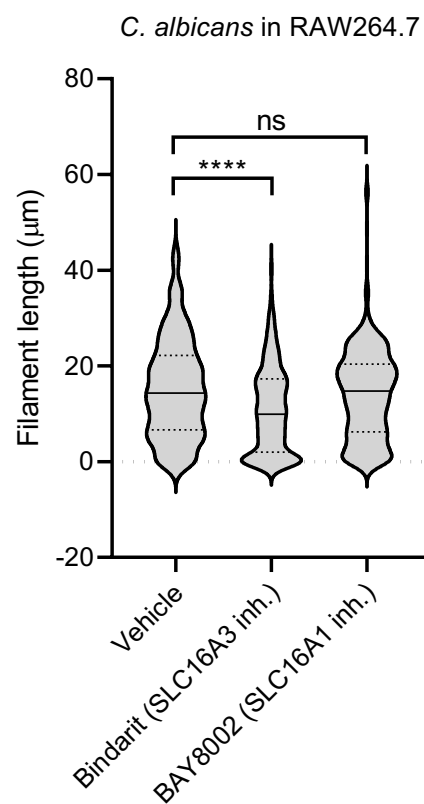**C**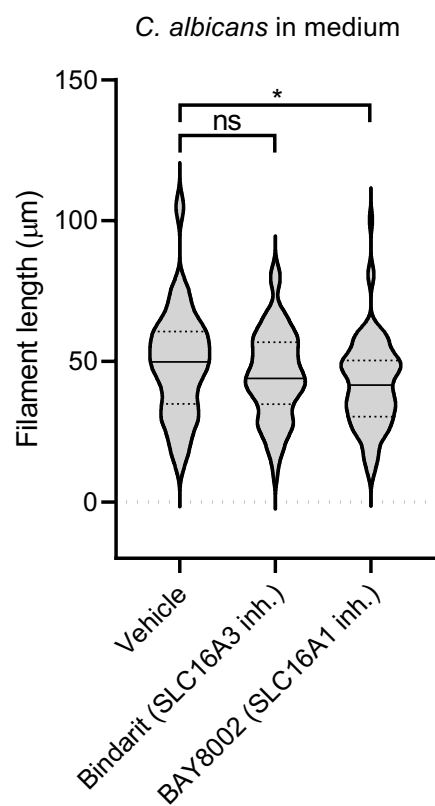**D**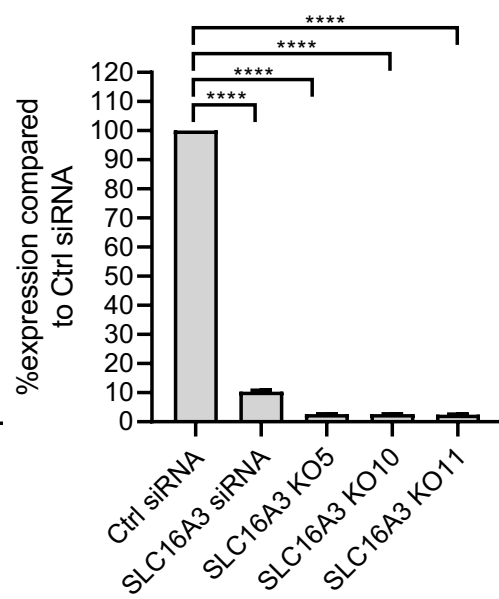**E**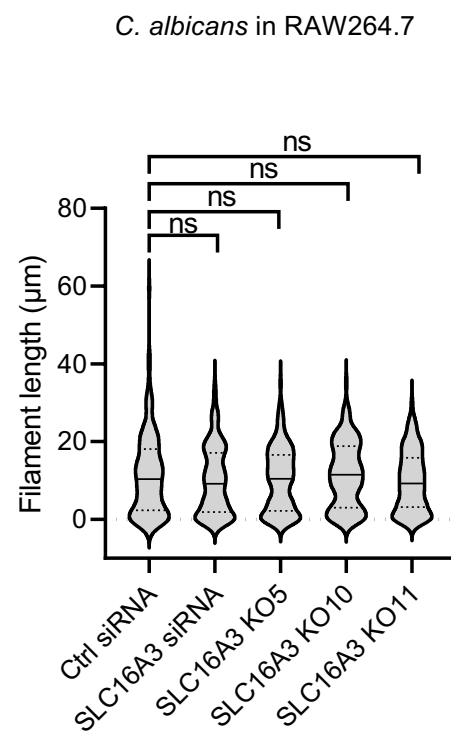

Supplement: Fig. S7 — Treatment of infected macrophages with BAY-876 or 2DDG does not decrease immune cell viability, and SLC16A3 is not essential for intraphagosomal filamentation of C. albicans. [file mbio.02745-23-s0008.pdf]

**A**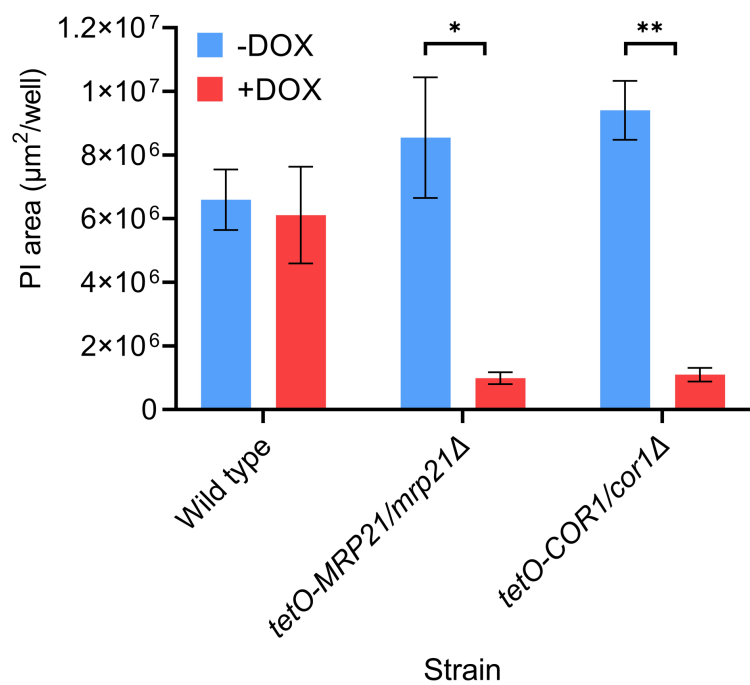**B**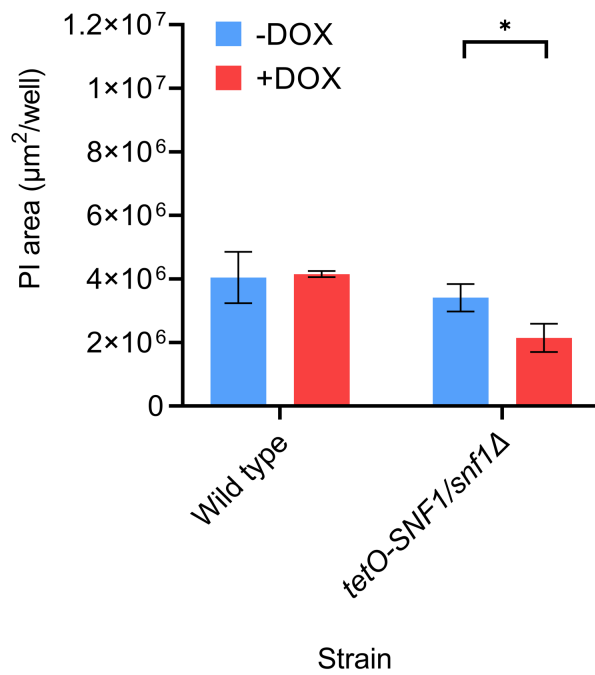

Supplement: Fig. S8 — Biological replicate of the experiment presented in Fig. 5D confirms reproducibility of results. [file mbio.02745-23-s0009.pdf]
