# Supplementary material for: Respiration supports intraphagosomal filamentation and escape of Candida albicans from macrophages
Source: mBio. 2023 Dec 1;14(6):e02745-23. doi: 10.1128/mbio.02745-23 (PMC10746240; doi:10.1128/mbio.02745-23)

A

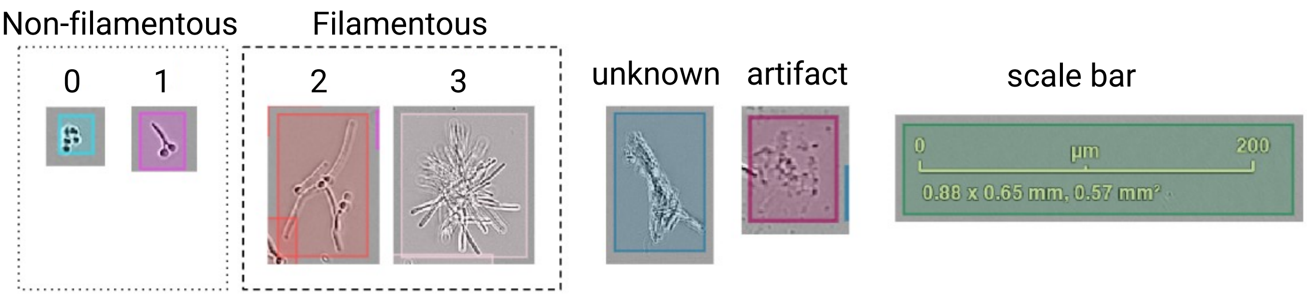

B

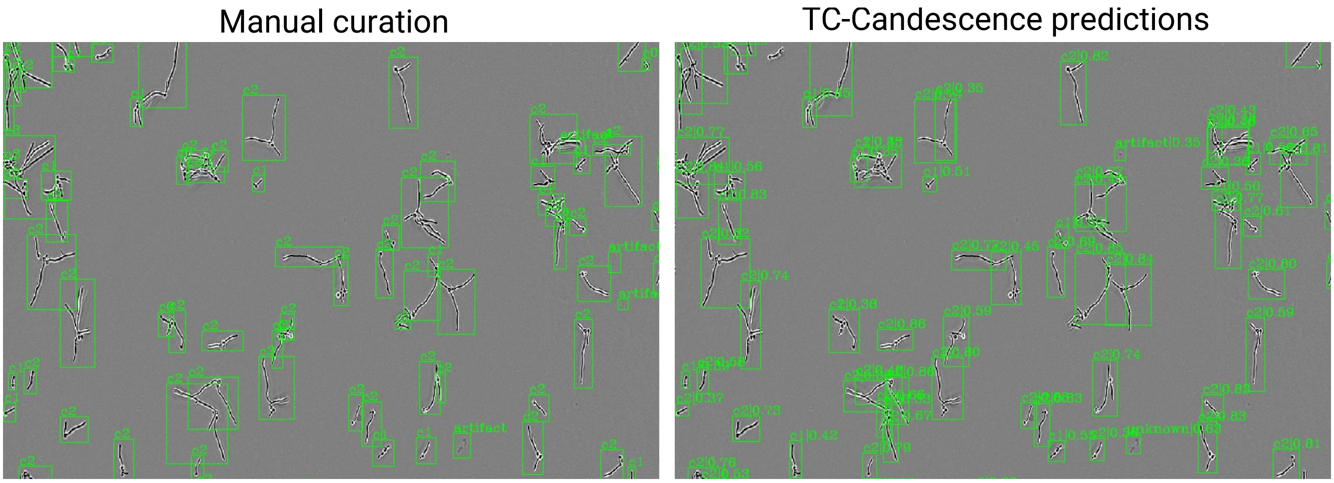

C

		Ground truth (manual curation)						
		non-filamentous		filamentous				
		0	1	2	3	unknown	artifact	Total
TC Prediction	0	105	40	3	0	28	4	180
	1	23	195	46	2	6	0	272
	2	1	69	584	21	7	0	682
	3	0	5	5	26	1	0	37
	unknown	12	2	3	0	28	6	51
	artifact	0	0	0	0	3	28	31
	Total	141	311	641	49	73	38	**1253**

No. correct classified: 966

No. classification errors: 287

false negatives/blindspots: 337

false positives/hallucinations: 216

Sensitivity: **0.746**

Precision: **0.664**

F1: **0.723**

D

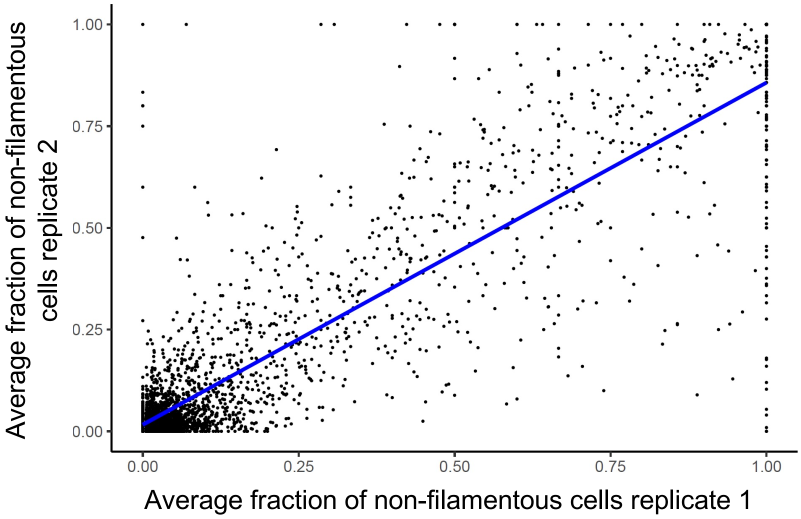

Supplement: Fig. S2 — Classification of C. albicans morphology under monoculture in TC conditions by TC-Candescence is sensitive and precise. [file mbio.02745-23-s0003.pdf]
